# Two Transcriptomic Analyses Converge on a Five-Gene Core for Colorectal Cancer Discrimination: External Validation in Independent Tissue Cohorts

**DOI:** 10.3390/genes17101177

**Published:** 2026-09-24

**Authors:** César de Jesús Tovar-Jácome, Luis Felipe Jave-Suárez, Jesús García-Gómez, Adriana Aguilar-Lemarroy, Augusto Sarralde-Delgado, Juan Manuel Carranza-Rosales, Jaime Patricio Barros-Núñez, Mónica Alejandra Rosales-Reynoso

**Affiliations:** 1 División de Medicina Molecular, Centro de Investigación Biomédica de Occidente (CIBO), Instituto Mexicano del Seguro Social (IMSS), Guadalajara 44340, Jalisco, Mexico; cesartovjacome@gmail.com (C.d.J.T.-J.); pbarros_gdl@yahoo.com.mx (J.P.B.-N.); 2 Doctorado en Genética Humana, Centro Universitario de Ciencias de la Salud (CUCS), Universidad de Guadalajara, Guadalajara 44340, Jalisco, Mexico; 3 División de Inmunología, Centro de Investigación Biomédica de Occidente (CIBO), Instituto Mexicano del Seguro Social (IMSS), Guadalajara 44340, Jalisco, Mexico; luis.jave@academicos.udg.mx (L.F.J.-S.); jesus.garcia9891@alumnos.udg.mx (J.G.-G.); 4 Doctorado en Ciencias Biomédicas, Centro Universitario de Ciencias de la Salud (CUCS), Universidad de Guadalajara, Guadalajara 44340, Jalisco, Mexico; 5 Unidad de Investigación Epidemiológica y en Servicios de Salud, Instituto Mexicano del Seguro Social (IMSS), Guadalajara 44340, Jalisco, Mexico; adry.aguilar.lemarroy@gmail.com; 6 Coordinador Auxiliar Médico de Investigación en Salud, Coordinación de Planeación y Enlace Institucional, OOAD Jalisco, Instituto Mexicano del Seguro Social (IMSS), Guadalajara 44340, Jalisco, Mexico; augusto.sarralde@imss.gob.mx or augusto.sarralde@gmail.com; 7 Coordinador Clínico de Educación e Investigación en Salud, OOAD Jalisco, Instituto Mexicano del Seguro Social (IMSS) Guadalajara 44340, Jalisco, Mexico; consultoriodrcarranza@gmail.com

**Keywords:** colorectal cancer, transcriptomic signature, RNA-seq, batch-effect correction, Elastic Net, machine learning, biomarkers

## Abstract

**Background/Objectives:** Colorectal cancer (CRC) is a leading cause of neoplasia-related morbidity and mortality worldwide and the first cause of cancer death in Mexico, where early detection remains a challenge, and reliable molecular biomarkers could support earlier diagnosis. Transcriptomic signatures for CRC have been published, but different studies arrive at gene lists that barely overlap, and since each study differs in both cohort and analysis, the source of that disagreement cannot be identified. This study aimed to identify a gene set for CRC discrimination whose selection depends neither on how the batch effect is handled nor on the criterion by which candidates are chosen and to validate that set in repositories that took no part in its selection. **Methods:** The signature was identified in 104 public RNA-seq samples (79 tumor, 25 reference) obtained from six BioProjects of the NCBI Sequence Read Archive and analyzed twice. A functionally anchored route took one representative gene per enriched biological program, ranked by π-value, from a differential expression model including BioProject as a covariate; a regularization route selected genes by their stability under Elastic Net in nested cross-validation over a ComBat-Seq-adjusted matrix. The genes reached by both routes were corroborated in TCGA-COAD, TCGA-READ, and GTEx colon processed by recount3 (*n* = 1542), with every parameter held fixed, and in the GEO microarray series GSE37364 (the 65 carcinoma and normal-mucosa samples of a 94-sample series), where only the coefficient vector was transferred because the platform differs; they were further examined descriptively in a second adenoma series (GSE89076), published stool RNA-seq, and plasma cell-free RNA. **Results:** The two routes yielded a seven-gene and a five-gene signature sharing a single gene, *RPGRIP1L*. Five genes met both criteria: *TRIP13*, *CEMIP*, *PUS7*, *RPGRIP1L*, and *SLC22A3*. With every parameter held fixed, the five-gene core reached an AUC of 0.9993 in TCGA/GTEx (sensitivity 0.966, specificity 0.998); with its coefficients transferred and the intercept recalibrated, it reached 0.9737 in GSE37364 (sensitivity 0.926, specificity 0.974). All five were elevated in adenoma relative to normal mucosa in GSE37364, and three of the five reached significance in GSE89076, where only five adenomas were available; all five were overexpressed in published stool RNA-seq of CRC patients. **Conclusions:** A five-gene core, *TRIP13*, *CEMIP*, *PUS7*, *RPGRIP1L*, and *SLC22A3*, discriminates colorectal tumor tissue from non-tumor in repositories external to its selection, with elevation already present in premalignant adenoma. Having been reached by two routes that share neither criterion nor starting point, these five genes are candidates for experimental validation and for evaluation in material accessible without biopsy.

## 1. Introduction

Colorectal cancer (CRC) ranks among the leading causes of neoplasia-related morbidity and mortality worldwide. In Mexico, it is the third most frequent cancer, with 15,697 new cases estimated for 2024, and the leading cause of cancer death, with 8539 deaths, 8.9% of all cancer deaths in the country [1]. Between 1998 and 2018, national mortality rose by 1.3% per year in women and 2.7% per year in men [2]. It represents a growing burden on health services, in part because diagnosis is frequently made at advanced stages [3]. Because survival depends largely on the stage at diagnosis, early detection remains the central unresolved problem in the clinical control of the disease [4].

Colonoscopy remains the gold standard for CRC detection, but its invasive nature, the bowel preparation it requires, and the need for sedation deter patient participation; adherence to screening therefore falls short of the 80% target [5]. Flexible sigmoidoscopy is better tolerated but examines only the distal colon, so proximal lesions fall outside its reach. Computed tomographic colonography reached a sensitivity of 64.9% and a specificity of 95.7% for colorectal neoplasia among participants with a positive fecal immunochemical test and still requires bowel preparation [6]. Among the non-invasive options, the fecal immunochemical test is the most widely used, although its sensitivity for CRC depends heavily on the positivity threshold applied: 67.3% at a cutoff of 20 µg of hemoglobin per gram of feces, with a specificity of 95.7% [7], and 96.5% at 10 µg/g, with a specificity of 89.0% [8]. Next-generation multitarget stool DNA testing reaches 93.9% sensitivity for CRC and 90.6% specificity for advanced neoplasia, at a per-sample cost some twenty times higher [7,8]. A cell-free DNA blood test evaluated in 7861 average-risk adults detected 83.1% of colorectal cancers with 90% specificity for advanced neoplasia, yet only 13.2% of advanced precancerous lesions [9], and blood-based methylation assays such as mSEPT9 are not recommended for screening on the basis of their performance [5]. Every non-invasive option trades sensitivity for accessibility, and the shortfall is widest for the lesions that precede invasive disease, as that 13.2% against 83.1% shows [9]. Molecular markers that add discriminatory information to the tests already in use and that raise the yield for premalignant lesions remain of interest.

The transcriptomic profile of a tumor serves as an alternative class of biomarker. A transcriptomic signature is a set of genes whose combined expression separates tumor from non-tumor tissue, and its discriminatory power comes from the pattern across genes rather than from any one of them. RNA sequencing measures that pattern without prior selection of targets, and public repositories now hold enough RNA-seq data to assemble cohorts larger than a single laboratory can produce [10]. When samples from different projects are analyzed together, they carry technical variability, known as the batch effect, which can be confounded with the biological signal [11]. Two approaches are in routine use. The first includes the project of origin as a covariate in the differential expression model, so that between-project variance is accounted for in the design [12]. The second adjusts the count matrix with ComBat-Seq before any model is fitted, so that subsequent analyses operate on corrected expression values [11]. Both are accepted practice, but they are not equivalent; Nygaard et al. showed that adjusting the matrix before modeling inflates confidence in downstream analyses relative to including batch in the design [13]. Whether the two also select the same genes is the question this study addresses.

More than forty multigene transcriptomic signatures have been published since 2007, and the field has moved from Affymetrix microarrays to RNA sequencing and, more recently, to single-cell and spatial approaches [14]. Panel sizes vary widely: ColoPrint uses 18 genes and ColoGuideEx 13 [15,16], while the Consensus Molecular Subtype classification was built from 4151 samples and defines four groups with prognostic value [17]. The panels share few genes with one another: an analysis of eight published CRC signatures found minimal overlap in gene composition and no functional agreement between them [18]. The disagreement also appears within one research group: feature selection over TCGA data identified *TMEM236* as a candidate diagnostic marker [19], and a later analysis by the same authors, over TCGA data and an additional series, arrived at *CA2*, *CA7* and *ITM2C* [20]. The two proposals share no gene. Different gene lists, and even randomly chosen ones, can be associated with the same outcome [21,22,23]; a signature obtained from a single cohort analyzed in a single way therefore says little about what a different analysis of the same samples would have selected.

Comparisons across published signatures cannot separate the contribution of the cohort from that of the method, because each study differs in both. Holding the samples constant and varying only the analysis removes that ambiguity: a gene selected under two treatments of the batch effect and two selection criteria owes its selection to neither. A single cohort of 104 colorectal tissue samples drawn from six public BioProjects was therefore analyzed twice, once with the BioProject entered in the model and once over a batch-adjusted matrix (Route A and Route B, Section 2), and the genes selected by both routes constitute the convergent core.

The aim of this study was to identify a gene set for colorectal cancer discrimination whose selection depends neither on how the batch effect is handled nor on the criterion by which candidates are chosen, and to validate that set in repositories that took no part in its selection. Validation used external tissue cohorts: TCGA-COAD, TCGA-READ, and GTEx colon through recount3 and the microarray series GSE37364. The behavior of the genes was further examined in premalignant adenoma, stool, and circulating plasma.

## 2. Materials and Methods

### 2.1. Study Design and Data Sources

A cross-sectional, analytical study was conducted on publicly available RNA-seq data from the NCBI Sequence Read Archive. BioProjects were screened against predefined criteria that can be verified in the run metadata of each project. Libraries had to be paired-end sequenced on an Illumina platform (Illumina, San Diego, CA, USA), LibrarySource had to be transcriptomic, and LibrarySelection had to be cDNA. Samples had to be colorectal tumor tissue or non-tumor colorectal tissue from the same project. Projects that provided only tumor tissue were included since reference tissue came from the remaining projects. Individual samples were retained when they reached 15 million reads and at least 94% of reads were mapped to GRCh38 with GENCODE v45 annotation.

Six BioProjects met these criteria: PRJNA1009137, PRJNA1055547, PRJNA596460, PRJNA913584, PRJNA394898 and PRJNA603221, which together provided 104 samples, 79 colorectal tumors and 25 reference tissues (Table 1). Four BioProjects provided both conditions; PRJNA1009137 and PRJNA394898 provided tumor tissue only. One project, PRJNA1009137, accounted for 48 of the 79 tumor samples, so BioProject and sample size were not evenly matched across the cohort. Tumor stage was available only for PRJNA1009137 and PRJNA1055547, which together covered 67 of the 104 samples; because it was missing for the other four projects, stage was not used as a stratification variable.

Further datasets were retrieved for external validation and took no part in gene selection or model fitting. Colorectal tumors from TCGA-COAD and TCGA-READ were combined with colon tissue from GTEx as processed by recount3 [24] (1542 samples: 669 tumor, 873 non-tumor). Two microarray series with adenomas were retrieved from GEO: GSE37364 (94 samples: 38 normal mucosa, 29 adenomas, 27 carcinomas) and GSE89076 (80 samples: 39 normal mucosa, 5 adenomas, 36 carcinomas). Stool RNA-seq results were taken from the supplementary data of Bahar Halpern et al. [25], and cell-free RNA data in plasma from exoRBase, whose source is given in Section 2.8. The way each of these datasets was used is described in Section 2.7 and Section 2.8. Three terms are used for non-tumor samples and are not interchangeable: reference tissue denotes the 25 non-tumor colorectal samples of the internal cohort, non-tumor tissue denotes the GTEx and TCGA samples that serve as controls in the external RNA-seq cohort, and normal mucosa denotes the colorectal mucosa of the microarray series, as labelled by the original studies.

### 2.2. Sequence Processing and Differential Expression

Reads were aligned and quantified in R with Rsubread, using GRCh38 with GENCODE v45 annotation, and gene-level counts were obtained with featureCounts [26,27]. Genes without at least 10 counts in at least three samples were removed. Differential expression was assessed with DESeq2 under a negative binomial generalized linear model that included BioProject as a covariate (~BioProject + Condition), so that the tumor-versus-reference effect was estimated with the technical variance between BioProjects accounted for in the model. Reference tissue was set as the control group, so that every log2 fold-change reported the change in tumor tissue relative to reference tissue [12]. Log2 fold change estimates were shrunk toward zero with the apeglm estimator [28], and *p*-values were adjusted by the Benjamini–Hochberg procedure. Genes with |log2 fold change| ≥ 1 and an adjusted *p*-value < 0.05 were considered differentially expressed. To check dependence on any single BioProject, the covariate model was refitted six times, each time without one BioProject, with the same filter and shrinkage, and the candidate genes obtained by the procedure described below were re-evaluated against the same criteria of direction of change, |log2 fold change| ≥ 1 and adjusted *p*-value < 0.05.

Candidate selection was restricted to overexpressed genes in both routes described below. The intended readout was a targeted assay, quantitative PCR, or immunohistochemistry, where an increase in signal over reference tissue was measured more reliably than a decrease; an under-expressed gene would need a different assay design.

A batch-adjusted expression matrix was generated separately with ComBat-Seq, which models raw counts under a negative binomial distribution while preserving the biological condition. Because DESeq2 accounts for BioProject through the design matrix, no adjusted values were required for differential expression; the ComBat-Seq matrix supplied adjusted per-sample expression for the regularization-based route and the models derived from it, as described in Section 2.4, Section 2.6, and Section 2.7.

### 2.3. Route A: Functionally Anchored Selection

Route A derived candidates from the functional characterization of overexpressed genes. Enrichment was performed on the 3018 overexpressed genes with pathfindR version 2.7.0 across Gene Ontology, KEGG, Reactome, and the Hallmark collection [29]. Protein–protein associations among the same genes were retrieved from STRING [30] and are reported as Supplementary Materials (Figure S1); they did not enter the selection.

Candidates were chosen under a rule fixed in advance. Enriched terms were grouped into non-redundant biological programs by shared function; each gene was assigned to a single program, the one containing its most significant term, and the gene with the highest π-value within each program was taken as its representative. The π-value combined effect size and statistical support as |log_2_ fold change| × −log_10_ (adjusted *p*) [31]. Representation by program was preferred to a global ranking by significance because term *p*-values scaled with the size of the annotated gene set and a global ranking therefore concentrated selection in the largest and most generic programs.

### 2.4. Route B: Selection by Stability Under Regularization

Independently of Route A, features were selected under a nested cross-validation design from the 2686 genes overexpressed on the ComBat-Seq-adjusted matrix (unshrunken DESeq2 log2 fold change ≥ 1, adjusted *p* < 0.05), transformed with the variance-stabilizing transformation of DESeq2 (blind to the design). In the outer loop, the samples were split into ten stratified folds; within each fold’s training partition an Elastic Net logistic regression model was fitted, and the standardized coefficient (β) of each selected gene was recorded [32]. The held-out fold was used only to generate out-of-fold predictions, in which each sample was predicted by a model trained without it. The L1/L2 mixing parameter and the regularization strength were tuned by an inner cross-validation within each training partition, so that fitting, tuning, and selection were confined to the training data of each outer fold.

Each gene was then summarized across folds by two quantities: its selection frequency, the fraction of the ten outer folds on which it received a non-zero coefficient, and its mean |β|, the average absolute coefficient over all ten folds, with unselected folds counted as zero. Genes selected in all ten folds formed the fully stable set.

Signature size was chosen within the same cross-validation. For each candidate size N (5, 10, 15, 20, and 30), a logistic regression was trained on the N genes with the largest Elastic Net coefficients in each training fold and scored on the held-out fold. The smallest N whose out-of-fold AUC was already near-maximal was retained, and the signature comprised that number of fully stable genes with the highest mean |β|.

### 2.5. Classification

Each gene panel was evaluated in a logistic regression classifier with L2-penalty, regularization fixed at C = 1.0, and class weights balanced to accommodate the 79:25 imbalance [33]. No hyperparameter search was performed, so no tuning information entered the evaluation.

Discrimination was summarized by the area under the ROC curve (AUC) and, given the class imbalance, by the area under the precision-recall curve (average precision), both computed from pooled out-of-fold predictions under stratified five-fold cross-validation, in which each sample was scored once by a model trained without it. Ninety-five percent confidence intervals (CI) were obtained from 1000 bootstrap resamples; each fitted on the resampled observations and scored on those left out. Significance against the null was assessed by 1000 label permutations scored under the same cross-validation scheme. Confusion matrices and the associated diagnostic metrics were computed on the pooled out-of-fold predictions at a fixed probability threshold of 0.50.

Each route was evaluated on the expression matrix produced by its own batch handling. The Route A representative signature was fitted on unadjusted log2 size-factor-normalized counts, since Route A accounted for BioProject within the differential expression model and left the expression values unaltered; the Route B signature and the convergent core were fitted on the variance-stabilized values of the ComBat-Seq-adjusted matrix described in Section 2.4, in which batch structure was removed from the values themselves. The log2 fold changes and adjusted p-values reported for the candidate genes (Section 3.5) come from the apeglm-shrunken DESeq2 results under each of the two batch-handling strategies, and their boxplots are drawn on the log2 (normalized count + 1) scale, whereas the constants of the convergent core classifier (Section 3.9) are on the VST scale.

### 2.6. Definition of the Convergent Core

The convergent core was defined as the intersection between the Route A representative signature and the genes selected in all ten outer folds of Route B. This fully stable set comprised every gene meeting Route B’s stability criterion. The Route B signature was a subset of it, reduced in size for parsimony (Section 2.4). Taking the intersection against the fully stable set therefore identified the genes retained by both routes, independently of the size chosen for that signature. The overlap between the two selections was compared against the hypergeometric expectation, taking the 2686 overexpressed genes as the universe.

### 2.7. External Validation and Coefficient Transfer

Two datasets that took no part in gene selection or model fitting were used to evaluate the convergent core. TCGA-COAD, TCGA-READ, and GTEx colon data (1542 samples: 669 tumor, 873 non-tumor) were obtained from recount3 [24], which processed both sources with a single pipeline, so alignment and quantification were uniform across origins. Per-gene differential expression was assessed with DESeq2 as in Section 2.2, with sample origin (TCGA-COAD, TCGA-READ, GTEx) as a covariate (~Origin + Condition); boxplots were drawn on origin-adjusted counts obtained with the removeBatchEffect function of limma [34]. For model transfer, expression was taken as log2 (size-factor-normalized count + 1). The second was GSE37364 (94 samples: 38 normal mucosa, 29 adenomas and 27 carcinomas; the 27 carcinomas and 38 normal-mucosa samples were used for classification) on the Affymetrix HG-U133 Plus 2.0 platform (Affymetrix, Santa Clara, CA, USA), as log2 intensities; probes were assigned to genes by discarding cross-hybridizing _x_at probe sets and retaining the probe set of highest mean intensity.

A logistic regression on the convergent core was trained once on the 104 internal samples using the batch-adjusted matrix, and its coefficients and intercept were then held fixed. Fitting on the adjusted matrix keeps the coefficients from absorbing variation specific to the internal BioProjects, which have no counterpart in the external cohorts. The transferred parameters are given in Section 3.9.

In TCGA/GTEx, every parameter was applied unchanged: the standardization constants, the coefficients, the intercept, and the threshold. No quantity was estimated in the target cohort, and the cross-validation carried out there only partitioned the samples for scoring. Expression in the target was standardized with the internal means and standard deviations, so the coefficients operate on the same standardized scale as in the internal fit.

In GSE37364, a different measurement platform, only the coefficient vector was transferred: expression was standardized with the target cohort’s own means and standard deviations, and the intercept alone was re-estimated so that mean predicted probability matched observed prevalence (calibration-in-the-large). This re-estimation used training folds only, so no held-out sample contributed to the intercept applied to it. Because the intercept entered the linear predictor as a constant shift, it could not alter the ranking of samples; the recalibration in GSE37364 therefore did not affect discrimination there. Both cohorts were evaluated under the protocol of Section 2.5.

### 2.8. Gene Behavior in Adenoma Series, Stool, and Plasma

Behavior of the convergent core was examined descriptively, without classification metrics, in two adenoma series, in stool and in plasma. In GSE37364, the 94 samples of the series (38 normal mucosa, 29 adenomas, 27 carcinomas) were compared as three groups; the two carcinoma stage groups of the original series (*n* = 14 and 13) were pooled since the present study did not address staging. GSE89076 (Agilent SurePrint G3 v2, Agilent Technologies, Santa Clara, CA, USA; 39 normal mucosa, 5 adenomas, 36 carcinomas, paired by patient) was retrieved with GEOquery [35] as log2 intensities, with probes assigned to genes as in Section 2.7 and *CEMIP* matched under its former symbol *KIAA1199*. In both series, groups were compared with the Kruskal–Wallis test followed by pairwise Wilcoxon tests with Benjamini–Hochberg adjustment; ties in microarray intensities were handled with the normal approximation. Log2 fold changes for tumor tissue and for stool, comparing colorectal cancer patients with healthy controls, and their adjusted *p*-values were taken from the supplementary data of Bahar Halpern et al. [25]. For plasma, the five-number summaries of cell-free RNA reported in exoRBase 2.0 [36] were used, and the genes without evaluable data in that resource are marked in the figure.

### 2.9. Ethical Considerations, Data Availability and Software

The study used publicly available data; the associated institutional protocol (R-2023-785-039) was classified as minimal risk under the Regulation of the General Health Law on Research Matters. The accessions of the analyzed BioProjects are provided in the data availability statement.

Read alignment and quantification, differential expression, batch correction, functional enrichment, retrieval of external datasets, and figure generation were carried out in R 4.4.3 using Rsubread 2.20.0, DESeq2 1.46.0 with apeglm 1.28.0, ComBat-Seq (sva 3.54.0), pathfindR 2.7.0, recount3 1.16.0, GEOquery 2.74.0, limma 3.62.2, and ggplot2 4.0.3. Protein–protein associations were retrieved from STRING v12.0. Classification, cross-validation, bootstrap, and permutation testing were carried out in Python 3.13.3 with scikit-learn 1.6.1 for the models and their evaluation, NumPy 2.3.4 and pandas 2.2.3 for data handling, SciPy 1.16.0 for statistical tests and Matplotlib 3.10.3 for figure visualization.

## 3. Results

The overall study workflow, from the integration of the public data to the external evaluation of the convergent core, is summarized in Figure 1.

### 3.1. Differential Expression in the Pooled Cohort

After low-count filtering, 37,420 genes were retained. With BioProject as a covariate, the comparison between tumor and reference tissue identified 6748 differentially expressed genes, 3018 overexpressed and 3730 under-expressed (Figure 2a). Principal component analysis separated tumor from reference samples along PC1 (30% of variance), and the sample-to-sample distance matrix recovered the same tumor-versus-reference structure by unsupervised clustering (Figure 2b,c). Within each condition, samples were grouped by BioProject, the technical structure that the two batch-handling strategies of Section 2.2 address.

### 3.2. Exploratory Functional Characterization

Enrichment of the overexpressed genes recovered a proliferative program across the four collections. Reactome returned activation of *ATR* in response to replication stress, G2/M and cell-cycle checkpoints, and DNA synthesis; KEGG returned DNA replication, cell cycle, and p53 signaling. The Hallmark collection returned E2F targets, *MYC* targets, and the G2/M checkpoint, with *CDK2*, *CDK4*, *CHEK1*, *MCM6*, *RFC3*, and *TRIP13* recurring across terms (Figure 3). Six further programs appeared with fewer genes each. Ribosome biogenesis was represented by rRNA processing and the fibrillar center (*NOP58*, *UTP20*, *DKC1*, *CEMIP*), tRNA metabolism by tRNA processing and pseudouridine synthase activity (*PUS7*, *XPOT*, *DKC1*), and the unfolded protein response by its Hallmark set (*DDX10*, *DKC1*, *XPOT*). Cilium assembly (*RPGRIP1L*, *CEP72*), transport of organic acids and metal ions (*SLC22A3*, *SLC6A6*, *SLC39A10*), and nuclear membrane and nuclear pore terms (*NUP107*, *IPO7*, *OSBPL3*) completed the picture. These enriched terms were grouped into the seven biological programs used for candidate selection in Section 3.3.

### 3.3. Route A Candidate Genes and Representative Signature

Functional enrichment of the overexpressed differentially expressed genes yielded terms that were grouped into seven biological programs, from which 26 genes were admissible (Table 2). The programs differed in size: proliferation and cell cycle accounted for 15 of the 26 genes, while rRNA processing and the nucleolus, tRNA processing, the unfolded protein response, ciliogenesis, solute transport, and the nuclear membrane contributed between one and three genes each.

### 3.4. Batch-Effect Adjustment with ComBat-Seq

The BioProject structure seen in Figure 2b was addressed a second time by adjusting the count matrix with ComBat-Seq while preserving condition. Before adjustment, PC1 captured 30% of the variance and separated BioProjects within each condition; after adjustment, PC1 rose to 44% and aligned with the tumor-versus-reference contrast, and the reference samples formed a single group (Figure 4). Differential expression on the adjusted matrix (~Condition) identified 7619 differentially expressed genes, 2511 overexpressed (2686 before apeglm shrinkage, the set that entered Route B) and 5108 under-expressed, against 6748 differentially expressed genes under the covariate model. The two strategies differed in how many genes crossed the thresholds. This adjusted matrix was the input of Route B (Section 3.7) and of the agreement check on the candidate genes (Section 3.5).

### 3.5. Agreement of the Candidate Genes Between Batch-Handling Strategies

The 26 candidate genes assigned to the seven biological programs (Table 2) were tested under both strategies. All 26 kept the same direction and remained significant with |log2 fold change| ≥ 1 under the BioProject-covariate model and on the ComBat-Seq-adjusted matrix (Table 3, Figure 5). Log2 fold changes ranged from 1.11 to 4.30 under the covariate model and from 1.03 to 4.25 on the adjusted matrix, with *CEMIP* the largest in both; the largest difference between strategies for any gene was 0.28 (*CEP72*). The candidate set therefore did not depend on how BioProject effects were handled, and the seven representatives chosen from it in Section 3.3 inherited that stability. Leaving out one BioProject at a time under the covariate model gave the same picture. The five genes of the convergent core met all three criteria in every exclusion (log2 fold change from 1.49 to 4.59), and 25 of the 26 candidates did so as well; *IPO7* kept its direction and significance but fell to a log2 fold change of 0.96 when PRJNA596460 was excluded. Excluding PRJNA1009137, which removed 48 of the 79 tumors and left 31 tumors against 25 reference samples, changed no verdict (Table S2).

### 3.6. Route A Representative Signature

The seven program representatives (Table 2) were evaluated as a predefined panel in the classifier described in Section 2.5. Thresholding the pooled out-of-fold predictions at 0.50 assigned 75 of 79 tumors and 24 of 25 reference samples correctly, giving a sensitivity of 0.949 and a specificity of 0.960, with the remaining operating-point metrics in Figure 6a. Ranking those same predictions rather than thresholding them gave a pooled AUC of 0.9863 (Figure 6b) and an average precision of 0.9954 (Figure 6c). The sampling variability of the AUC was estimated from 1000 bootstrap resamples (95% CI 0.9483–1.0000; Figure 6d), and the observed value fell outside the null distribution of 1000 label permutations (*p* < 0.001; Figure 6e).

Standardized coefficients ranged from −0.64 to +1.51 (Figure 6f). *TRIP13* (+1.51), *CEMIP* (+1.43), and *SLC22A3* (+1.15) carried the largest weights, followed by *DKC1* (+0.42) and *OSBPL3* (+0.20). Two genes received negative coefficients, *RPGRIP1L* (−0.10) and *PUS7* (−0.64); both were overexpressed in tumor tissue (Table 2).

### 3.7. Route B Signature

Independent of the Route A candidates, Elastic Net selected genes directly from the matrix of 2686 overexpressed genes across the ten cross-validation folds. Each gene’s selection frequency was the fraction of folds retaining it, and its mean |β| the average magnitude of its coefficient across folds. Twenty-one genes were retained in every fold and occupy the top of the Figure 7 heatmap, with the largest mean |β| in the right-hand panel. The forty most stable genes are displayed.

To determine how many of these stable genes were needed to separate tumor versus reference, classifiers of increasing size were compared by out-of-fold AUC (Figure 8). Because the genes entering each model were chosen inside the training fold, each held-out fold was unseen at both the selection and the fitting step. The full Elastic Net reached an out-of-fold AUC of 0.999 (95% CI 0.997, 1.000), and fixed-size models matched it from five genes onward, holding between 0.998 and 1.000 up to thirty genes. The smallest five-gene signature was retained for parsimony, *RPGRIP1L*, *ETV4*, *BMAL2*, *PPM1H*, and *AJUBA*, the five stable genes with the highest mean |β|.

The five-gene signature was then evaluated as a fixed panel in the same classifier used for Route A (Figure 9). Those genes were selected using all 104 samples, and the cross-validation reused those same samples, so this AUC is an optimistic estimate. It served as the matched comparison with Route A, while the nested 0.999 remained Route B’s primary estimate. At the 0.5 threshold, the model correctly assigned all 79 tumors and 23 of 25 reference samples, giving a sensitivity of 1.000 and a specificity of 0.920 (Figure 9a). The pooled out-of-fold AUC was 1.000 (Figure 9b) and the average precision 1.000 (Figure 9c). Every bootstrap resample returned an AUC of 1.000, so the resulting interval was degenerate and did not describe sampling variability (Figure 9d). The observed AUC fell outside the null distribution of 1000 label permutations (*p* < 0.001; Figure 9e). All five standardized coefficients were positive (Figure 9f): *RPGRIP1L* (1.41), *ETV4* (1.21), *BMAL2* (1.01), *PPM1H* (0.98), and *AJUBA* (0.48).

### 3.8. Convergent Core

The two final signatures shared one gene, *RPGRIP1L*, and their union comprised eleven distinct genes. The overlap was larger at the level of stable selection: five of the seven Route A representatives, *TRIP13*, *CEMIP*, *PUS7*, *RPGRIP1L*, and *SLC22A3*, were also retained by Route B in all ten folds, and these five constituted the convergent core (Table 4). The remaining two representatives, *DKC1* and *OSBPL3*, were not among Route B’s stable genes. If the two routes selected independently among the 2686 overexpressed genes, the expected overlap between the 7 Route A representatives and the 21 genes retained by Route B in all ten folds would be 0.05 genes; 5 were observed (hypergeometric *p* = 3.6 × 10^−10^). The two routes are not fully independent, since both start from overexpressed genes, so this figure bounds the surprise rather than testing it formally.

### 3.9. External Validation of the Convergent Core

The convergent core was evaluated in two cohorts that took no part in its selection. A logistic regression on the five genes was trained once on the 104 internal samples and held fixed; its coefficients and standardization constants are given in Table 5.

In TCGA/GTEx, the model of Table 5 was applied without re-estimating any parameter. Thresholding its predictions at the transferred cut-off of 0.50 produced the confusion matrix and operating-point metrics in Figure 10a: 646 of 669 tumors and 871 of 873 non-tumor samples correct, giving a sensitivity of 0.9656 and a specificity of 0.9977. Ranking the same predictions rather than thresholding them, the ROC curve in Figure 10b reaches a pooled AUC of 0.9993, and the precision–recall curve in Figure 10c reaches an average precision of 0.9992. The bootstrap distribution in Figure 10d places the 95% confidence interval of the AUC at 0.9985–0.9999, and the permutation test in Figure 10e locates the observed value outside the null distribution of 1000 label shuffles (*p* < 0.001). The decision surface in Figure 10f shows the two groups falling on opposite sides of the 0.50 probability contour.

In GSE37364, the same coefficient vector was applied to the target cohort, with the intercept re-estimated as described in Section 2.7. At the 0.50 threshold, Figure 11a reports 25 of 27 carcinomas and 37 of 38 normal-mucosa samples correctly assigned, a sensitivity of 0.926, and a specificity of 0.974. The ROC curve in Figure 11b gives a pooled AUC of 0.9737, and the precision–recall curve in Figure 11c an average precision of 0.9750. The bootstrap distribution in Figure 11d places the 95% confidence interval of the AUC at 0.9286–1.0000, its upper bound at unity, reflecting the small number of samples available for resampling, and the permutation test in Figure 11e again separates the observed value from the null distribution of 1000 label shuffles (*p* < 0.001). The decision surface in Figure 11f reproduces the separation seen in the RNA-seq cohorts on a hybridization platform.

### 3.10. Behavior of the Convergent Core in Adenoma Series, Stool and Plasma

The five genes were examined descriptively in two adenoma series, in stool and in plasma, without classification metrics (Section 2.8). In GSE37364 (38 normal mucosa, 29 adenoma, 27 carcinoma), all five were elevated in adenoma relative to normal mucosa (Wilcoxon, BH-adjusted *p* from 4.8 × 10^−3^ for *RPGRIP1L* to 2.9 × 10^−13^ for *CEMIP)* and in carcinoma relative to normal mucosa (from 3.0 × 10^−2^ for *RPGRIP1L* to 1.2 × 10^−16^ for *CEMIP*). Between carcinoma and adenoma, only *TRIP13* differed (adjusted *p* = 0.0018); *CEMIP*, *PUS7*, *RPGRIP1L*, and *SLC22A3* did not (adjusted *p* 0.46, 0.23, 0.42, and 0.67) (Figure 12a). GSE89076 (39 normal mucosa, 5 adenoma, 36 carcinoma; *CEMIP* annotated under its former symbol *KIAA1199*) reproduced the pattern with five adenomas: *CEMIP*, *TRIP13*, and *PUS7* were elevated in adenoma relative to normal (adjusted *p* 0.022, 0.013, and 0.0019); *RPGRIP1L* and *SLC22A3* changed in the same direction without reaching significance (0.16 and 0.26); all five separated carcinoma from normal (from 1.6 × 10^−7^ to 8.5 × 10^−13^); and again only *TRIP13* separated carcinoma from adenoma (0.013) (Figure 12b).

In stool, the RNA-seq comparison of colorectal cancer patients against healthy controls reported by Bahar Halpern et al. [25] detected the five genes, all overexpressed in patients: log2 fold change 3.16 for *CEMIP*, 2.09 for *RPGRIP1L*, 2.01 for *SLC22A3*, 1.53 for *PUS7*, and 1.41 for *TRIP13*. The same study measured tumor tissue, where the values were 3.07, 2.10, 1.84, 1.82, and 1.91, so the stool signal tracked the tissue signal gene by gene (Figure 13a). That cohort contained no adenoma group. In plasma cell-free RNA (exoRBase), *TRIP13* and *PUS7* had no evaluable signal, and *CEMIP* and *RPGRIP1L* had lower medians in patients than in healthy controls (0.42 versus 0.75 and 0.09 versus 0.20), the opposite direction to tissue and stool. *SLC22A3* had a median of zero in both groups, with the third quartile falling from 0.60 in healthy controls to 0.30 in patients. All three differences were reported as *p* < 0.01 by the database (Figure 13b).

## 4. Discussion

The present study sought to identify a gene set for colorectal cancer discrimination whose selection survives a change in both the treatment of the batch effect and the criterion of selection. The same 104 samples were analyzed by a functionally anchored route, which took one representative gene from each enriched biological program, and by a regularization route, which selected genes according to their stability under Elastic Net regularization, each fitted on the expression matrix produced by its own batch handling. Both approaches distinguished tumor from reference tissue in internal cross validation, with a pooled AUC of 0.986 for the Route A panel and a nested estimate of 0.999 for Route B, and their final signatures shared one gene. The principal contribution of the study is the subset of genes on which the two approaches agreed: their identification by strategies of markedly different design reduces the likelihood that they reflect an artifact of a single analytical pipeline and that set retained its discrimination in two cohorts that took no part in selecting it.

Route A fixed which genes were eligible before any classifier was fitted, and it did so on marginal evidence; each representative was the gene with the highest π-value in its program, a quantity computed for one gene at a time. Nothing in that rule examines how the selected genes behave together. Fitted jointly, the seven representatives redistributed their weights accordingly (Figure 6f): *TRIP13*, *CEMIP*, and *SLC22A3* carried most of the discrimination, while *RPGRIP1L* and *PUS7* received negative coefficients, although both were overexpressed in tumor tissue (Table 2). A coefficient in a multivariate model measures what a gene contributes once the others are present, so among predictors carrying overlapping information, its sign need not follow the direction of the gene’s own association. The negative weights are a property of the panel rather than evidence against the genes, and they mark what a criterion that never evaluates genes jointly leaves undetermined. Biological relevance and predictive necessity are therefore not equivalent: a gene may be strongly and reproducibly associated with the tumor phenotype yet add little, or enter with the opposite sign, once other genes already carry its discriminatory information.

The central finding lies in the five genes on which the two routes converged. *TRIP13*, *CEMIP*, *PUS7*, *RPGRIP1L*, and *SLC22A3* were program representatives in Route A and were retained by Route B in all ten outer folds, so their association with the tumor phenotype satisfies two criteria that share neither their starting point nor their notion of evidence. The intersection was taken against the genes retained in every fold rather than against Route B’s final signature, so the core does not depend on where the size of that signature was cut; two representatives, *DKC1* and *OSBPL3*, did not meet the stability criterion.

The strength of this result stems from the different nature of the two tests these genes passed. Route A ranks each gene on its own association with the phenotype and on the biological program it represents, one gene at a time. Route B asks whether a gene keeps a non-zero coefficient when it is penalized alongside every other candidate, and whether it does so regardless of which samples train the model, on a matrix from which the project structure has been removed rather than absorbed by a design term. Neither criterion contains the other; a gene with the strongest marginal evidence in its program can be dropped once its neighbors enter the model, and a gene retained in all ten folds need not be the most significant member of any program. The five genes satisfy both, so their selection does not depend on which of the two routes was taken, the source of disagreement that comparisons between published signatures cannot isolate, since those studies differ in cohort and in method at once.

The convergent core was then carried into data that took no part in its selection. In TCGA/GTEx, the model was applied with its standardization constants, coefficients, intercept, and threshold all fixed, so no quantity was estimated in the target cohort. It assigned 646 of 669 tumors and 871 of 873 non-tumor samples correctly, with a pooled AUC of 0.9993. The constants derived from the internal adjusted matrix therefore placed the target samples on a comparable scale without adjustment. What this shows is that separation is not confined to the cohort in which the genes were selected. It does not show that the magnitude of the AUC reflects tumor biology alone.

GSE37364 subjects the same panel to a further change. Its intensities come from a hybridization array and are not on a scale comparable to log_2_ counts, so only the coefficients were transferred, and the intercept alone was re-estimated; since a constant shift could not reorder samples, the ranking remained that of the transferred model. The panel reached an AUC of 0.9737, assigning 25 of 27 carcinomas and 37 of 38 normal-mucosa samples correctly, so the discrimination survived a change of quantification technology as well as of cohort. Neither of these estimates reused the samples in which the genes were chosen, which is what distinguishes them from every internal figure reported here.

The five genes of the convergent core have functions congruent with tumor biology. *TRIP13*, an AAA+ ATPase of the spindle assembly checkpoint, is overexpressed in colorectal tumors, promotes proliferation and invasion through its interaction with *YWHAZ*, and is an independent predictor of poor survival [37]; *CEMIP* degrades the extracellular matrix and has been linked to the Wnt/β-catenin pathway and to metastatic progression in CRC [38,39]; *PUS7* catalyzes RNA pseudouridylation, has been proposed as a prognostic biomarker in multiple tumors, and in CRC, its expression has been linked to proliferation and metastatic capacity [40,41]. *SLC22A3* encodes the organic cation transporter OCT3, which mediates cellular uptake of oxaliplatin; in colorectal cancer, higher OCT3 expression has been associated with longer progression-free survival under oxaliplatin-based adjuvant chemotherapy [42]. That association comes from a cohort annotated with treatment and outcome, which the present design does not include. *RPGRIP1L*, linked to primary cilia assembly, is the least characterized of the five in colorectal cancer and the gene on which the two routes agree most closely: it is the only member of both final signatures and carries the largest weight in the Route B model and in the transferred core (Table 5). In the present data, it is consistently overexpressed in tumor tissue (Table 2), retained in every fold of Route B, and positively weighted in the model that transferred to both external cohorts; its negative partial coefficient in the Route A panel (Figure 6f) follows from the composition of that panel rather than from its direction of change. It has been reported as downregulated in hepatocellular carcinoma, where it was proposed as a candidate tumor suppressor [43], a tumor type and tissue distinct from the one examined here. What its overexpression reflects in colorectal tissue has not yet been established, and it is presented as a candidate for functional study rather than with an assigned role [44].

Comparison with published signatures is bound by their purpose. ColoPrint, ColoGuideEx, and Oncotype DX colon [15,16,45] were built to predict recurrence and shared no genes with the 26 candidates or the Route B signature. Among tumor-versus-mucosa signatures, Maurya et al. [19,20] shared none either, and Lee et al. [46] shared *CEMIP*, a Route A candidate, among their prioritized genes, and *ETV4*, form the Route B signature, among the ten they evaluated (Table S1). The absence of overlap with the prognostic signatures was expected; the small overlap among tissue classifiers is the situation the present design was meant to address since each of those studies differ from the others in both cohort and method. What the present analysis contributes is a gene set reached by two routes that share neither their criterion nor their starting point and validated in a cohort of 1542 RNA-seq samples and in an independent microarray series.

Two Route A representatives, *DKC1* and *OSBPL3*, were not retained by Route B in every fold. Each was the strongest marginal candidate of its program, but neither held a non-zero coefficient across all resamples once fitted alongside the remaining candidates, so their association with the tumor phenotype rests on a single criterion. Both are nonetheless reported in colorectal cancer: *DKC1*, whose product dyskerin participates in rRNA pseudouridylation and telomerase assembly, is elevated in colorectal tumors, promotes angiogenesis through HIF-1α transcription, and is associated with metastasis and shorter survival [47]; *OSBPL3* is elevated in tumor relative to normal mucosa and is an independent prognostic marker in multi-omics analyses [48].

On the other hand, the four genes unique to Route B were reached by stable selection over the overexpressed genes, without passing through the functional criterion. Each has been reported in colorectal cancer. *ETV4*, an ETS-family transcription factor, promotes epithelial-mesenchymal transition and metastasis, and its silencing reduces tumor proliferation [49,50,51]. *AJUBA* acts on the Hippo/YAP pathway and favors proliferation [52,53]. *BMAL2 (ARNTL2*), a circadian-clock gene, is overexpressed in colorectal tumor tissue and has been linked to worse prognosis and to resistance to 5-fluorouracil [54,55]. *PPM1H*, a PP2C-family phosphatase, has a context-dependent role; both oncogenic overexpression and association with relapse at low expression have been reported [56,57]. All four rest on a single route and require independent confirmation. Route B’s fully stable set extends beyond its signature and includes *ATP11A* and *SLC6A6* (Table 4), both already reported in colorectal cancer: *ATP11A* maintains the asymmetry of membrane lipids, and its disruption promotes cell motility and invasion [58,59], a mechanism also reported in other tumors through the inactivation of the Hippo pathway [60]; *SLC6A6* transports taurine and contributes to tumor survival, chemoresistance and CD8^+^ T-cell mediated immune evasion [61,62]. Neither was retained as a program representative, so neither enters the core.

The first set of limitations concerns the performance estimates. The internal metrics describe separability within this dataset rather than generalization to new patients, and they are optimistic for identifiable reasons. The genes of each fixed panel were chosen using all 104 samples before the cross-validation that scored them, so Figure 6 and Figure 9 measure how well those panels separate the samples that produced them. The nested estimate of Figure 8 avoids that reuse. What it estimates is the selection procedure, with no particular panel behind those numbers, and per-fold values of 1.000 are what a separable problem returns when ten or eleven samples are held out. The Route B figures carry one further caveat, since supervised analysis of a corrected matrix can exaggerate apparent separation in a design where some BioProjects contribute tumor samples only [13]. These concerns fall on the metrics, not on the identity of the genes; Route A’s differential expression was computed on raw counts with BioProject as a covariate, so the program representatives and the convergent core do not depend on the corrected matrix, and the external cohorts passed through no correction of ours. The transferred model was scored on values produced by pipelines outside this study and separated the groups there as well. The post-correction principal component analysis (Figure 4) is likewise a quality check rather than evidence of discrimination since separation along the condition axis is expected once that condition has been preserved during correction. The magnitude of these AUCs should not be read as the result of the study. Tumor tissue and non-tumor colorectal mucosa are transcriptionally distant states, and near-ceiling discrimination is what most reasonable gene sets achieve on that contrast; the figures reported here fall where that contrast usually puts them. Three factors push them upward in a predictable order. The internal panels were chosen on the same 104 samples that the cross-validation then scored, so Figure 6 and Figure 9 measure how separable those samples are and say nothing about new ones. In TCGA/GTEx, the tumor and the non-tumor samples were contributed by two different programs, and recount3 quantified all 1542 through one pipeline, so the cohort of origin and condition still vary together. GSE37364 removes both: the gene took no part in its selection, and cases and controls were collected in one study, on one platform, under one protocol. The AUC there is 0.9737, lower than the 0.9993 of TCGA/GTEx, and that ordering is what the two designs predict. The claim the study makes rests on which genes two different selection criteria agreed upon, not on how high the classifier scores; the classifier is the instrument used to rank and confirm those genes, not the product being proposed.

A second set of limitations concerns design and the cohorts. Candidate selection was confined to overexpressed genes in both routes, which sets the scope of what the study can find. The intended endpoint is detection with targeted assays, where a gain of signal relative to reference tissue is the quantity that can be measured reliably, so the agreement reported here is agreement within that set and says nothing about genes under-expressed in tumor tissue. The analysis set was drawn from six public BioProjects differing in population and protocol, two of which contributed tumor samples only, so the tumor-versus-reference contrast is not available within every project. Leaving out each BioProject in turn, including the one that contributes 48 of the 79 tumors, left the 26 candidates and the convergent core within the selection criteria (Section 3.5, Table S2), so the candidate set is not the signature of any single project. In TCGA/GTEx, the non-tumor samples come from a different study than the tumors, so that comparison, like the internal one, sets tumor against non-tumor colorectal tissue, never against matched adjacent mucosa from the same patient. The 79:25 imbalance was handled by class weighting, and TCGA/GTEx, where non-tumor samples outnumber tumors, tests the model under the opposite proportion. Both external cohorts are public repositories rather than prospectively collected series, so confirmation in samples gathered under a clinical protocol is still needed. Stage was available for 67 of the 104 samples and was not used, so the design says nothing about early or advanced disease; experimental validation of the signatures is underway but lies beyond the scope of this work.

The classifier used here is the instrument by which the genes were identified and ranked, not the intended product. Separating a resected tumor from reference tissue is not a question that requires a transcriptomic model, and the value of the core lies in material where the diagnosis is not already established. Section 3.10 examined the two settings that matter for that use. In premalignant disease, where blood-based assays detect the majority of established carcinomas but a small minority of advanced precancerous lesions [9], the five genes were already elevated in adenoma relative to normal mucosa in GSE37364, and three of them (*CEMIP*, *TRIP13*, and *PUS7*) in the five adenomas of GSE89076; in both series only *TRIP13* differed further between carcinoma and adenoma.

The pattern is that of a signal present from the premalignant lesion onward, which is the property a detection marker requires and the opposite of what a staging marker would show. In stool, the five transcripts were detected and overexpressed in patients with colorectal cancer in the series of Bahar Halpern et al. [25], with fold changes that tracked those of tumor tissue in the same study; in plasma cell-free RNA, *TRIP13* and *PUS7* had no evaluable signal, *CEMIP* and *RPGRIP1L* were lower in patients than in controls, and *SLC22A3* showed no increase, so the plasma compartment does not reproduce the tissue direction and is not a candidate material for these transcripts on present evidence. These comparisons are descriptive and carry no classification metric. The link the present data cannot close is whether the transcripts are elevated in stool from patients with adenoma since the stool series contains no adenoma group; that measurement, and the addition of any such marker to the fecal immunochemical test, are the next steps.

On the biological side, the five genes were reached by discrimination rather than by pathway membership, and they represent distinct programs: proliferation, nucleolar and rRNA processing, tRNA modification, ciliogenesis, and solute transport. The same separation appears in their STRING neighborhood (Figure S2). Of the ten interactors in the first shell, six belong to RPGRIP1L and form the MKS complex of the ciliary transition zone, three to TRIP13, among them MAD2L1BP, its partner in spindle checkpoint silencing, and one to PUS7. CEMIP and SLC22A3 have none, and no interactor links two core genes.

The core is a set of genes that separate each tumor from reference under two selection criteria, without evidence that they act together as a module. What connects them in colorectal tissue, if anything does, is an open question rather than a result of this work, and *RPGRIP1L* is the member for which functional characterization would be most informative, being the least described in this tissue while carrying the largest weight in the transferred model. The immediate practical step remains confirmation in a dedicated Mexican cohort, which would test the genes in the target population and in prospectively collected samples, followed by their integration into targeted expression assays and their measurement in stool, where the diagnostic question remains open. Whether the same transcripts also carry information beyond that contrast, about stage, prognosis, or response to treatment is a separate question, one the present design cannot address since it compares tumor with non-tumor tissue only; cohorts annotated with clinical outcome would be needed to pursue it.

## 5. Conclusions

Two methodologically distinct bioinformatics approaches, applied to the same 104 samples under different treatments of the batch effect, yielded two transcriptomic signatures for CRC, each distinguishing tumor from reference tissue in internal cross-validation. The two signatures share a single gene, but five were reached by both criteria: *TRIP13*, *CEMIP*, *PUS7*, *RPGRIP1L*, and *SLC22A3* were program representatives in the functionally anchored route and were retained in every fold of the regularization route. With the same coefficients applied in both, these five separated tumors from non-tumor tissue in 1542 RNA-seq samples and in an independent microarray series, neither of which took part in their selection. In adenoma, the five genes were elevated relative to normal mucosa in one series and *CEMIP*, *TRIP13*, and *PUS7* in a second, and in published stool RNA-seq, all five were overexpressed in patients with colorectal cancer. This agreement, achieved by two approaches of different design and sustained outside the data in which it was found, is the principal result and positions these five genes as priority candidates for experimental validation and for evaluation in material accessible without biopsy.

## Figures and Tables

**Figure 1:**
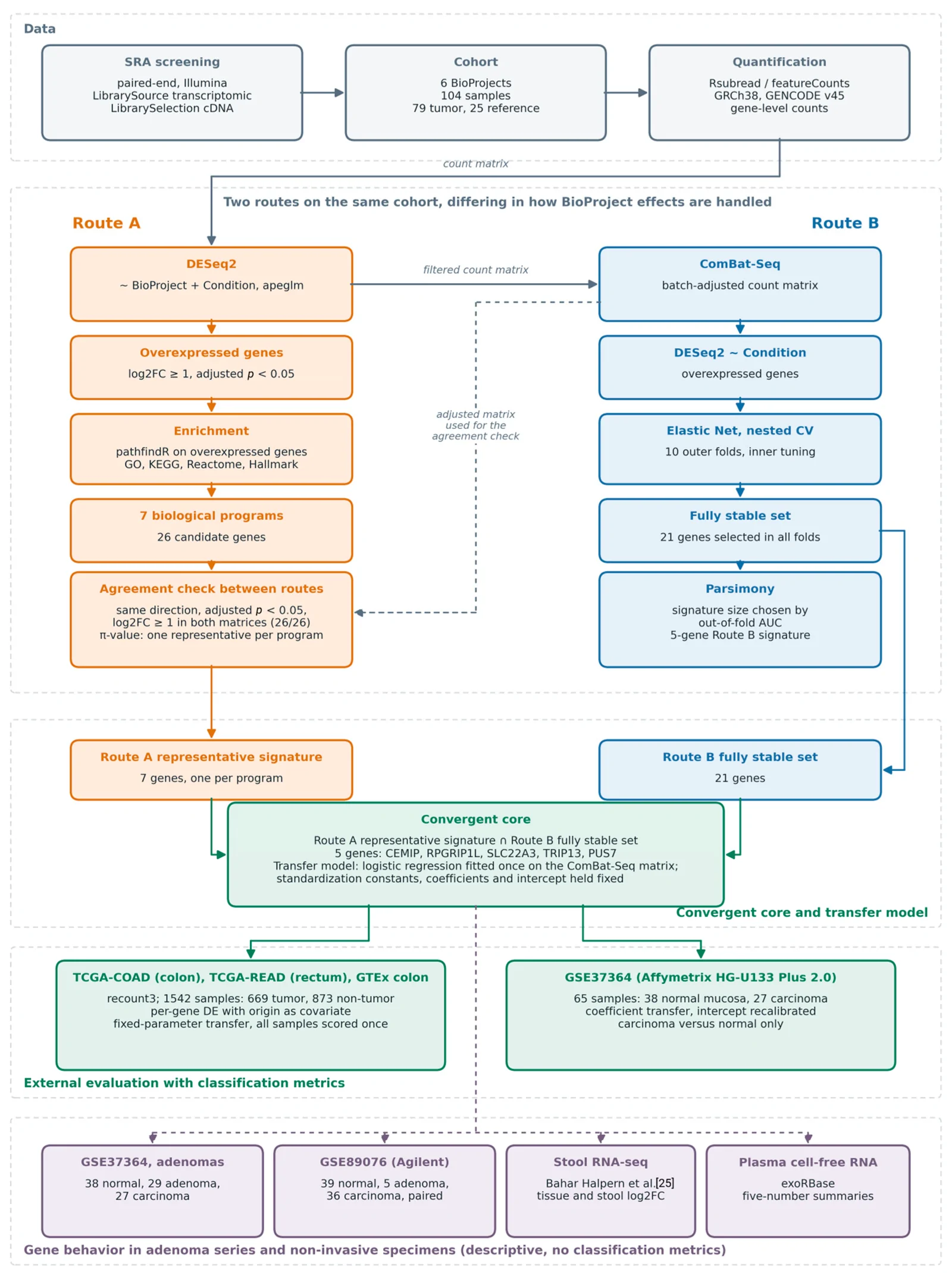
Study workflow. A single cohort of 104 paired-end samples from six SRA BioProjects (79 tumor, 25 reference) was analyzed through two routes that differ in how BioProject effects are handled. Route A modeled BioProject as a covariate in DESeq2 and selected one representative per biological program by π-value; the candidate genes were then checked for agreement between the two batch-handling strategies. Route B applied ComBat-Seq to the same filtered count matrix and selected genes by stability under Elastic Net. The convergent core was the intersection of the Route A representative signature with the fully stable set of Route B; its transfer model was trained once on the 104 internal samples and held fixed. External evaluation with classification metrics used TCGA-COAD, TCGA-READ, and GTEx colon (fixed-parameter transfer) and the normal mucosa and carcinoma samples of GSE37364 (coefficient transfer). The adenomas of that same series, the paired series GSE89076, stool and plasma were examined descriptively. Internal metrics were cross-validated estimates on the same 104 samples that produced the signatures, and external metrics came from cohorts that took no part in selection or fitting.

**Figure 2:**
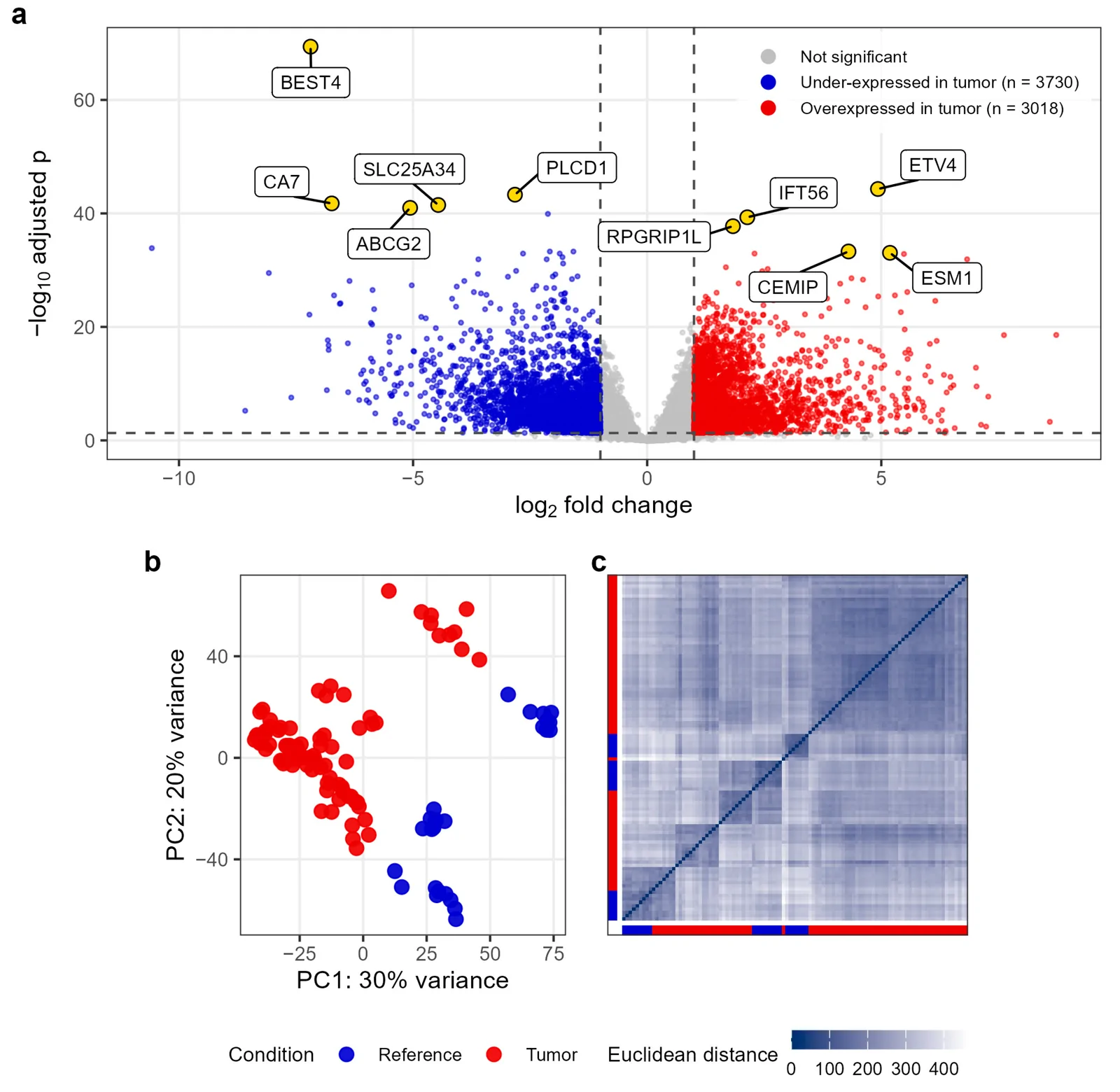
Differential expression and sample structure in the pooled cohort (*n* = 104). (**a**) Volcano plot of DESeq2 results with BioProject as a covariate and apeglm shrinkage. Of the 37,420 genes tested, the 37,369 with an adjusted *p*-value are shown. Red, overexpressed in tumor (*n* = 3018); blue, under-expressed in tumor (*n* = 3730); grey, not significant. Dashed lines mark |log2 fold change| = 1 and adjusted *p* = 0.05; the five genes with the lowest adjusted *p*-value in each direction are labeled. (**b**) Principal component analysis on variance-stabilized counts of the 500 most variable genes, without batch adjustment; PC1 30% and PC2 20% of variance. Samples group by BioProject within each condition, the technical structure that the covariate model accounts for. (**c**) Sample-to-sample Euclidean distance matrix on the same values, ordered by hierarchical clustering; side bars indicate condition. Reference tissue in blue and tumor in red throughout.

**Figure 3:**
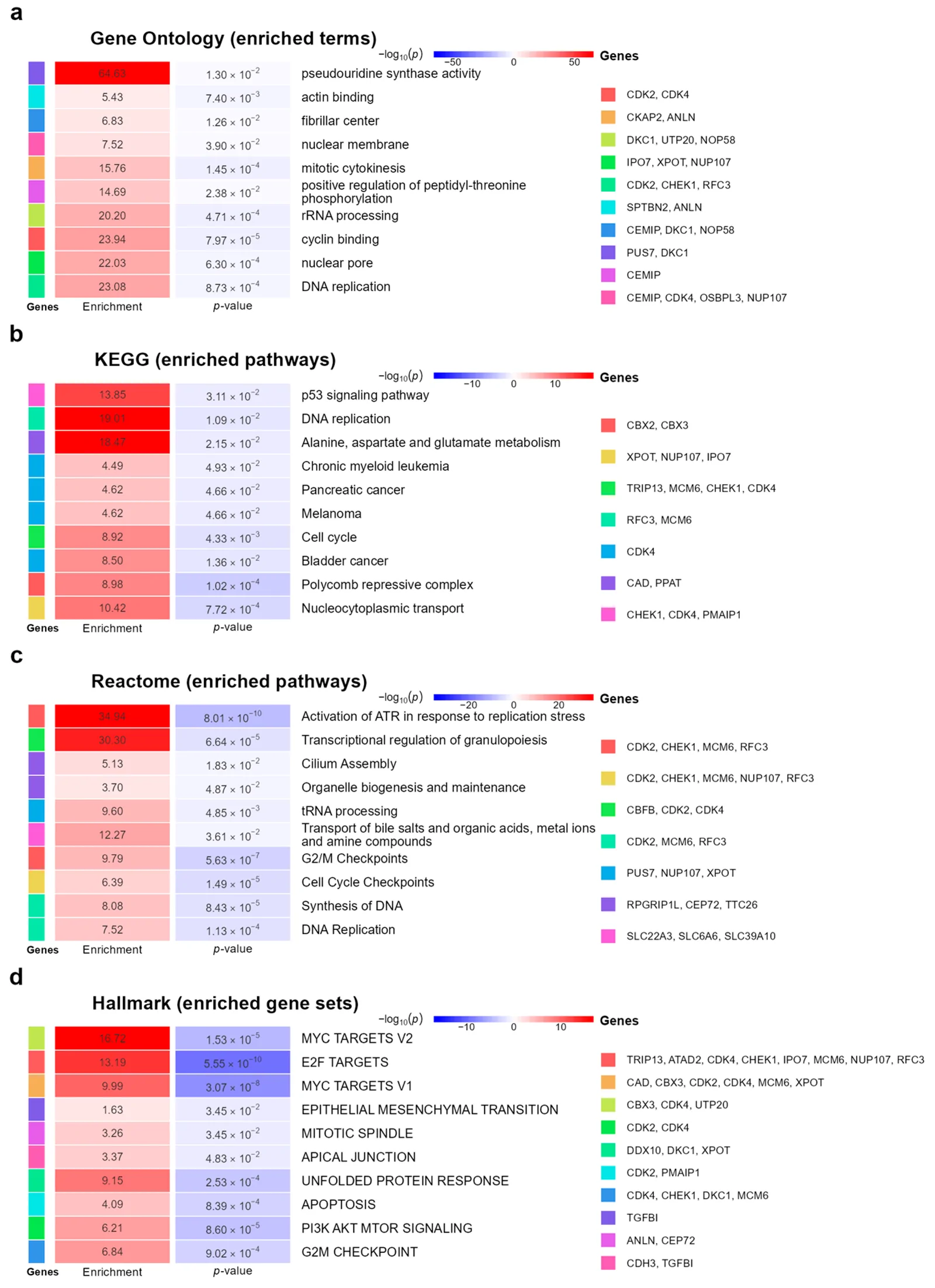
Functional enrichment of the overexpressed genes in the pooled cohort (pathfindR). (**a**) Gene Ontology, (**b**) KEGG, (**c**) Reactome, and (**d**) Hallmark collections. Rows are enriched terms; the left strip marks the contributing genes, the first column gives fold enrichment and the second the adjusted *p*-value, both color-coded by −log_10_ (adjusted *p*).

**Figure 4:**
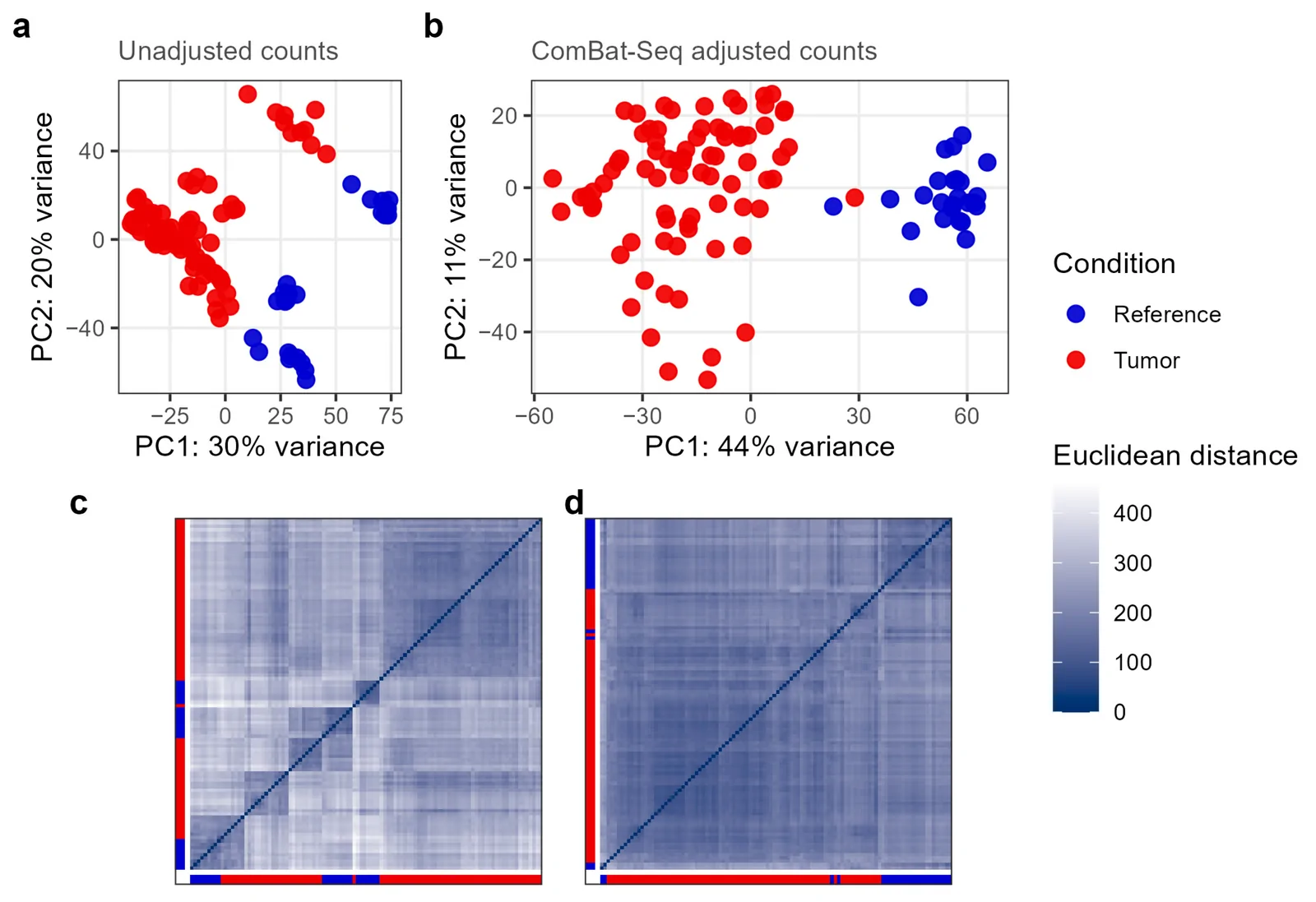
Effect of ComBat-Seq adjustment on the sample structure. Principal component analysis of the 500 most variable genes on variance-stabilized counts before (**a**) and after (**b**) ComBat-Seq, and the corresponding sample-to-sample Euclidean distance matrices (**c**,**d**); the diagonal of each matrix is the zero distance of each sample to itself. The unadjusted counts are the input of the model with BioProject as a covariate (Route A), and the counts adjusted with ComBat-Seq are the input of Route B. Reference tissue in blue and tumor in red.

**Figure 5:**
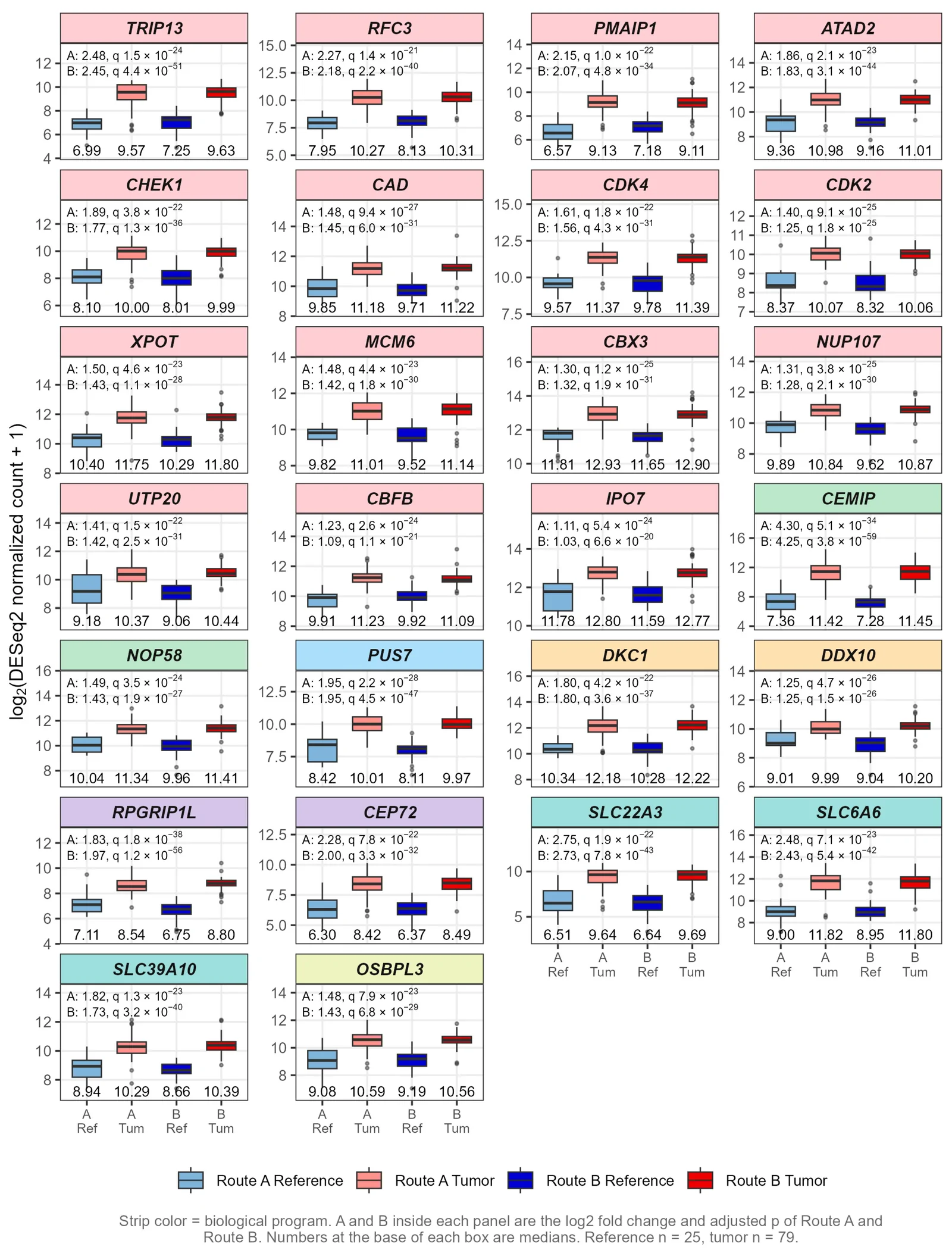
The 26 candidate genes under the two batch-handling strategies. Boxplots of log2 (DESeq2 normalized count + 1) in reference (blue) and tumor (red) tissue for each gene, under Route A (size-factor normalization, BioProject entered as a covariate; light shades) and Route B (ComBat-Seq-adjusted counts; dark shades). Panels are grouped by biological program (strip color). Numbers at the base of each box are medians; the annotation in each panel gives the DESeq2 log2 fold change (apeglm) and adjusted *p* (q) for tumor versus reference under each route. All 26 genes keep the same direction and log2 fold change ≥ 1 under both strategies; values are listed in Table 3. Reference *n* = 25, tumor *n* = 79.

**Figure 6:**
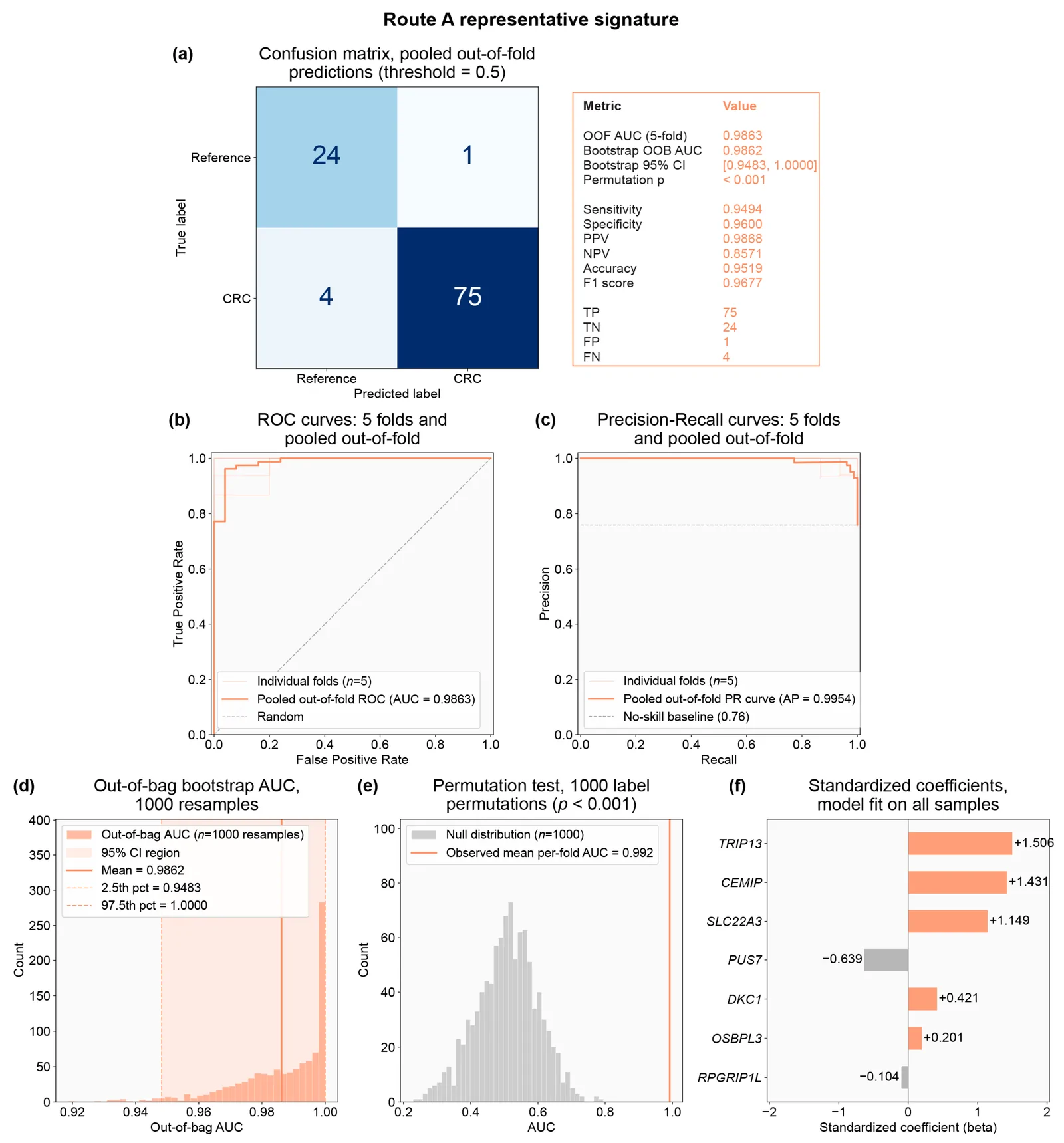
Route A representative signature: classification performance of the seven program representatives (*TRIP13*, *CEMIP*, *PUS7*, *DKC1*, *RPGRIP1L*, *SLC22A3*, and *OSBPL3*) in a logistic regression classifier under stratified five-fold cross-validation. (**a**) Confusion matrix of the out-of-fold predictions at a 0.50 probability threshold, with the associated metrics table. The AUC (0.9863) was derived from the ranked out-of-fold probabilities and was threshold-independent; sensitivity (0.9494), specificity (0.9600), PPV (0.9868), NPV (0.8571), accuracy (0.9519), F1, and likelihood ratios were evaluated at the 0.50 threshold. (**b**) ROC curves for the five individual folds and for the pooled out-of-fold predictions; the pooled AUC (0.9863) was derived from the ranked probabilities and was therefore threshold-independent. The dashed diagonal marks the performance of a random classifier. (**c**) Precision–recall curves for the five folds and for the pooled out-of-fold predictions (pooled AP = 0.9954), which summarize performance under the 79:25 class imbalance. The dashed line marks the tumor prevalence of 0.76, the no-skill baseline under this class proportion. (**d**) Out-of-bag AUC across 1000 bootstrap resamples (mean 0.9862; 95% CI 0.9483–1.0000), showing the sampling variability of the AUC. (**e**) Permutation test: the observed cross-validated AUC relative to the null distribution from 1000 label permutations (*p* < 0.001). (**f**) Standardized logistic-regression coefficients.

**Figure 7:**
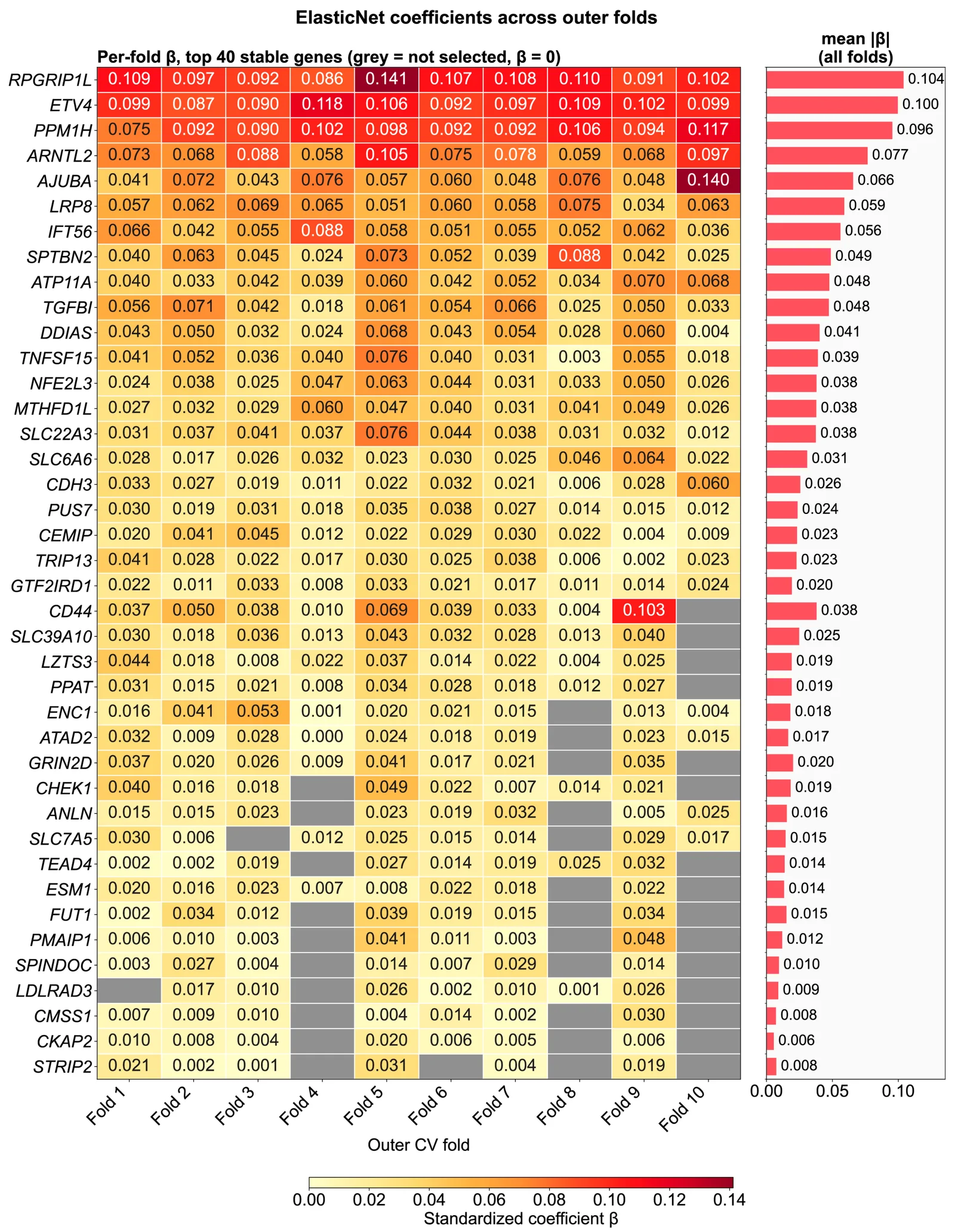
Elastic Net stable selection across ten stratified cross-validation folds. The heatmap shows per-fold standardized coefficient (β) of the most stable genes, ordered by mean |β| across folds (right panel); grey cells mark folds in which a gene was not selected (β = 0), which count as zero in the mean. The forty most stable genes are shown; twenty-one were selected in all ten folds.

**Figure 8:**
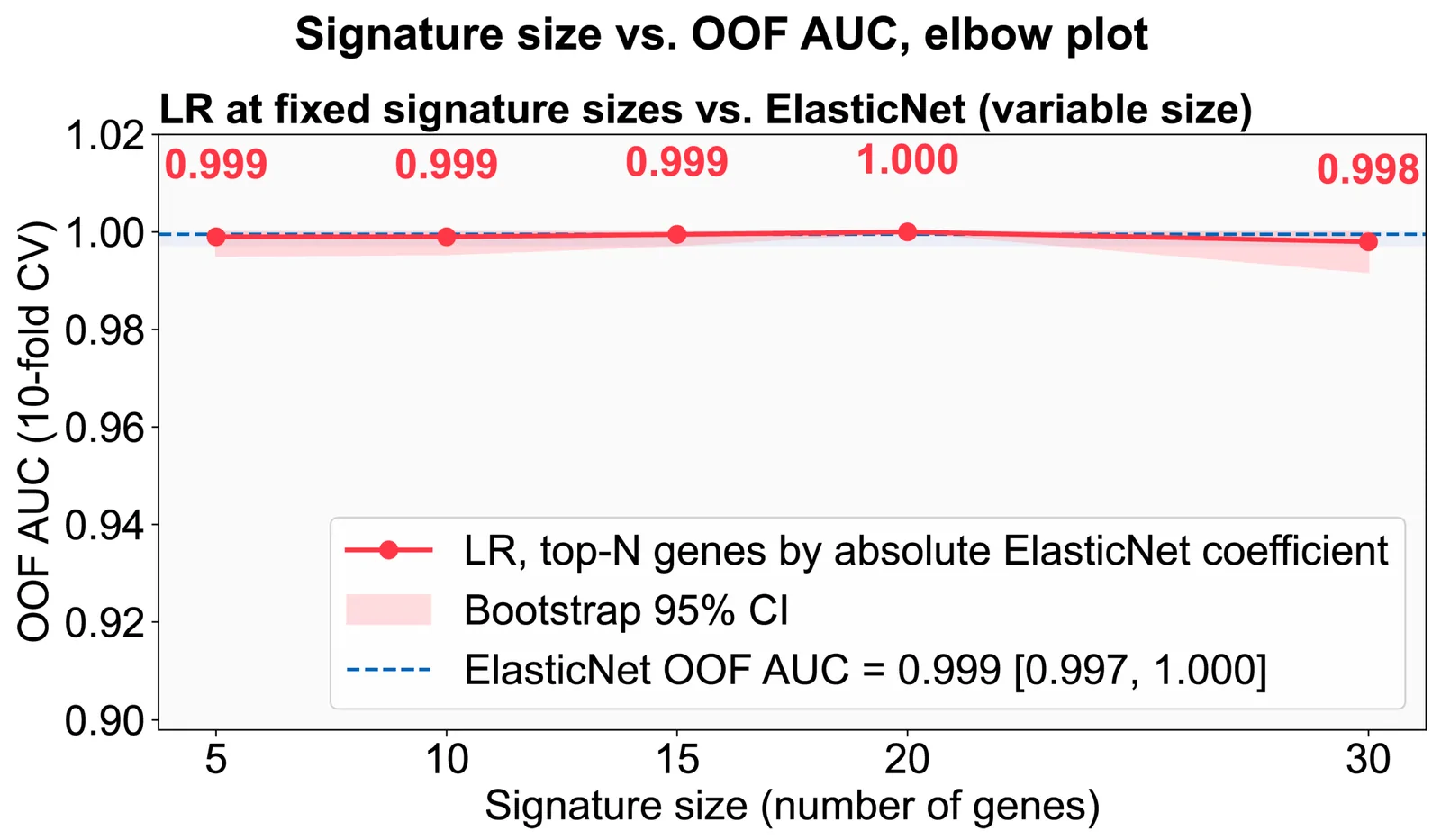
Signature size versus out-of-fold classification performance (Route B). For each fixed size N (5, 10, 15, 20, 30), a logistic regression was trained on the N genes with the largest absolute Elastic Net coefficients in each training fold and scored on the held-out fold; the red curve shows the resulting out-of-fold AUC in ten-fold cross-validation, and the shaded band its bootstrap 95% confidence interval. The blue dashed line marks the out-of-fold AUC of the full, variable-size Elastic Net (0.999; 95% CI 0.997–1.000) for reference. Performance is near-maximal from five genes onward and does not improve as more are added, supporting the five-gene signature on grounds of parsimony.

**Figure 9:**
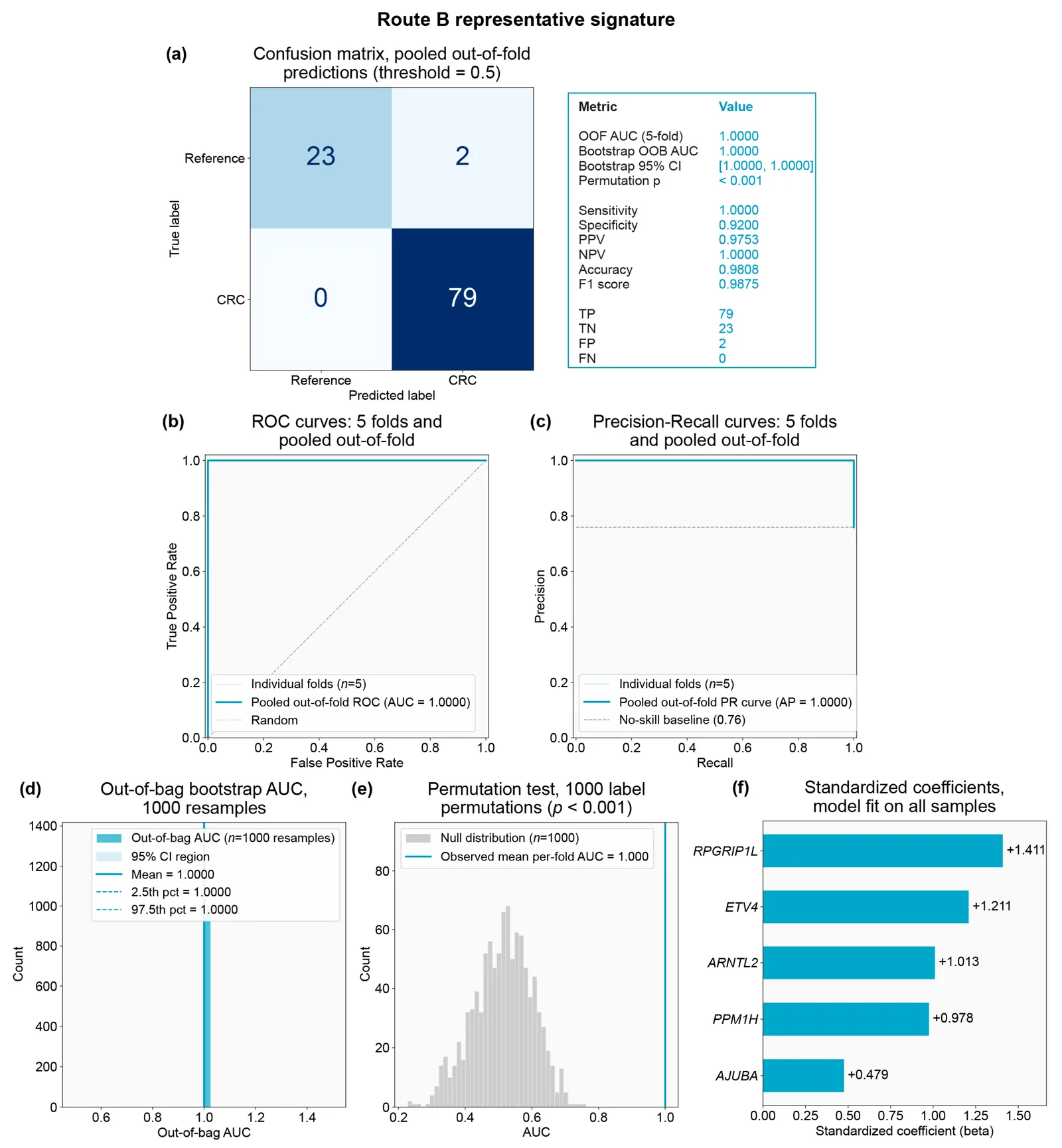
Route B signature: classification performance of the five stable genes (*RPGRIP1L*, *ETV4*, *BMAL2*, *PPM1H*, and *AJUBA*), in a logistic regression classifier under stratified five-fold cross-validation, evaluated as a fixed panel for comparison with Route A. (**a**) Confusion matrix of the out-of-fold predictions at a 0.5 probability threshold, with the associated metrics table; sensitivity (1.000), specificity (0.920), PPV (0.975), NPV (1.000), accuracy (0.981), F1, and likelihood ratios were all evaluated at that threshold. (**b**) ROC curves for the five individual folds and for the pooled out-of-fold predictions; the pooled AUC (1.000) was derived from the ranked probabilities and was therefore threshold-independent. The dashed diagonal marks the performance of a random classifier. (**c**) Precision–recall curves for the five folds and for the pooled out-of-fold predictions (pooled AP = 1.000). (**d**) Out-of-bag AUC across 1000 bootstrap resamples (mean 1.000; 95% CI 1.000 to 1.000). The dashed line marks the tumor prevalence of 0.76, the no-skill baseline under this class balance. (**e**) Permutation test: the observed AUC lies entirely outside the null distribution from 1000 label permutations (*p* < 0.001). (**f**) Standardized logistic-regression coefficients, all positive.

**Figure 10:**
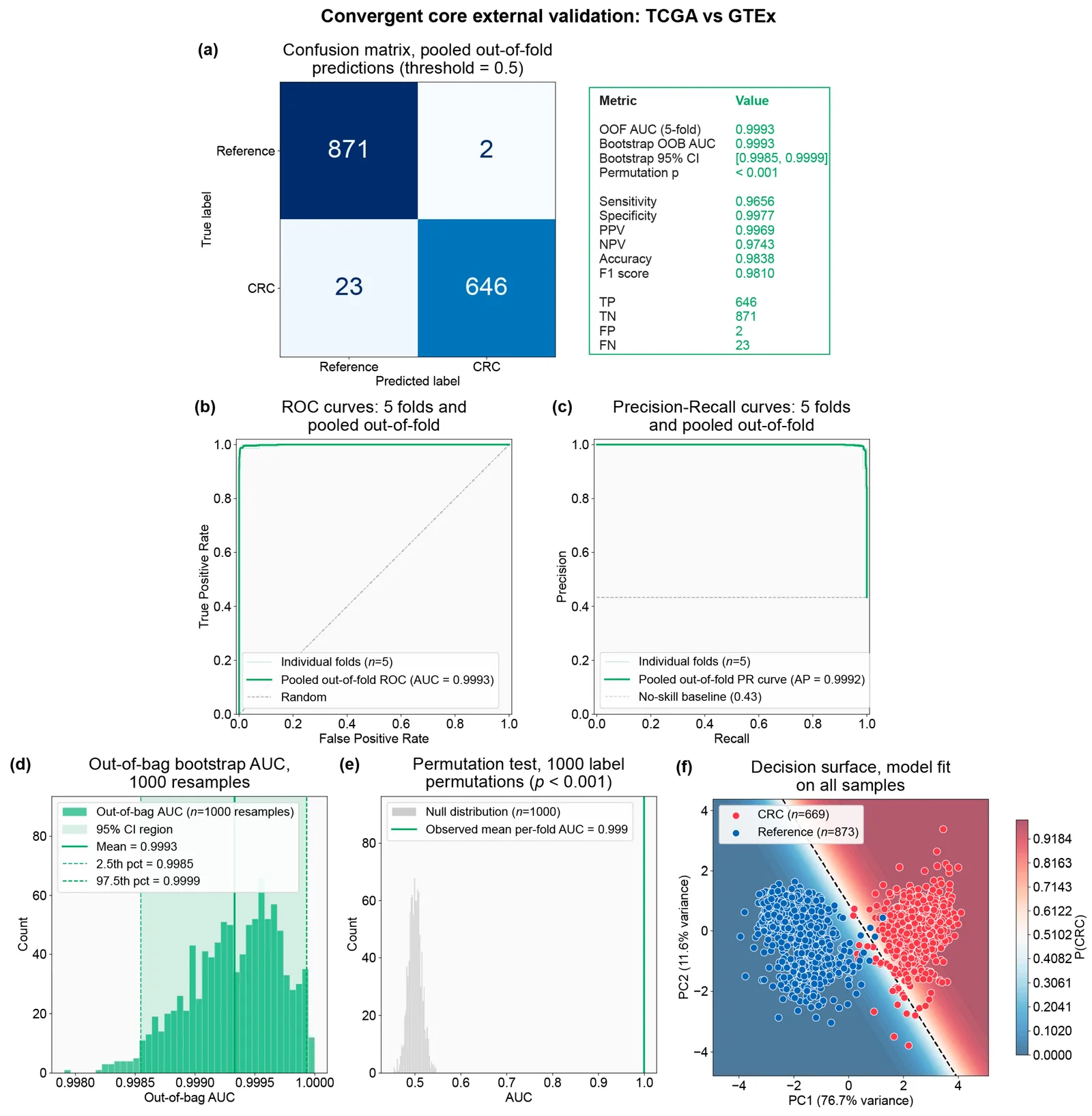
Convergent core, external validation in TCGA/GTEx (1542 samples; 669 tumor, 873 non-tumor). The five-gene model of Table 5 was applied with all parameters unchanged, standardization constants, coefficients, intercept, and threshold, so no quantity was estimated in this cohort. (**a**) Confusion matrix at the 0.50 probability threshold, with the associated metrics table; PPV (0.9969), NPV (0.9743), accuracy (0.9838), F1, and likelihood ratios were evaluated at that threshold. (**b**) ROC curves for the individual cross-validation folds and for the pooled predictions; the pooled AUC was derived from the ranked probabilities and was therefore threshold-independent. The dashed diagonal marks the performance of a random classifier. (**c**) Precision–recall curves for the individual folds and for the pooled predictions. The dashed line marks the tumor prevalence of 0.434, the no-skill baseline under this class balance. (**d**) Out-of-bag AUC across 1000 bootstrap resamples (mean 0.9993). (**e**) Null distribution of the area under the curve over 1000 permutations of the diagnostic labels, with the observed value marked (*p* < 0.001). (**f**) Decision surface of the transferred model, projected onto the first two principal components; the dashed contour marks a predicted probability of 0.50.

**Figure 11:**
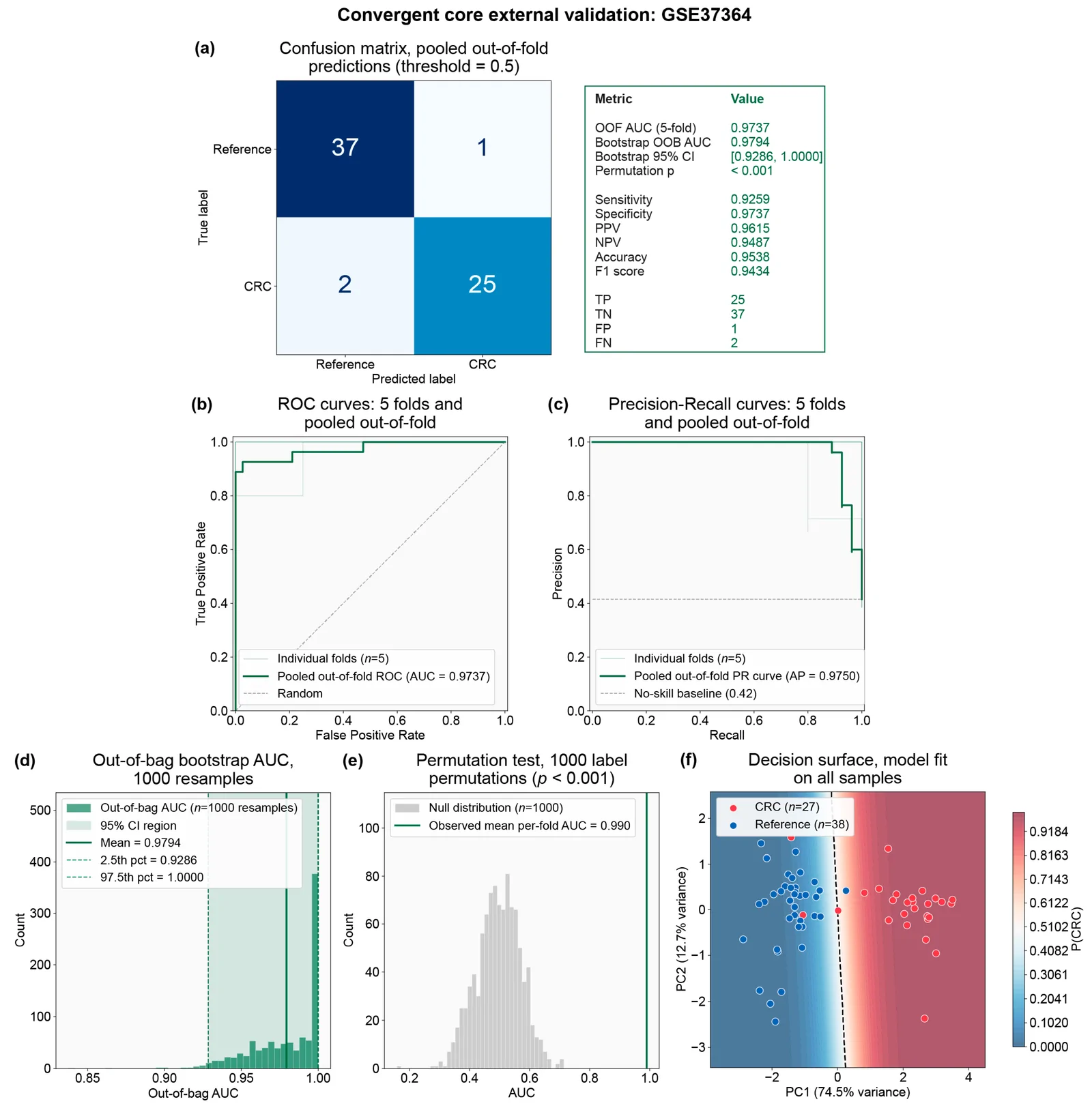
Convergent core external validation: GSE37364. Independent evaluation of the five-gene convergent core in the 65 carcinoma and normal mucosa samples of GSE37364, a 94-sample series profiled on Affymetrix HG-U133 Plus 2.0 (27 colorectal carcinoma, 38 normal mucosa), a cohort that took no part in gene selection or model fitting. The coefficient vector of Table 5 was transferred unchanged and the intercept re-estimated by calibration-in-the-large. (**a**) Confusion matrix at the 0.50 probability threshold, with positive predictive value 0.962, negative predictive value 0.949, accuracy 0.954 and F1 0.943. (**b**) Receiver operating characteristic curve. The dashed diagonal marks the performance of a random classifier. (**c**) Precision–recall curve; the dashed line marks the carcinoma prevalence of 0.415 expected of a no-skill classifier. (**d**) Distribution of the area under the curve over 1000 bootstrap resamples, with the observed value and the 2.5th and 97.5th percentiles indicated. (**e**) Null distribution of the area under the curve over 1000 permutations of the diagnostic labels, with the observed value marked (*p* < 0.001). (**f**) Decision surface of the transferred model, projected onto the first two principal components; points are individual biopsies, red for carcinoma and blue for normal mucosa, and the contour marks the 0.50 probability boundary.

**Figure 12:**
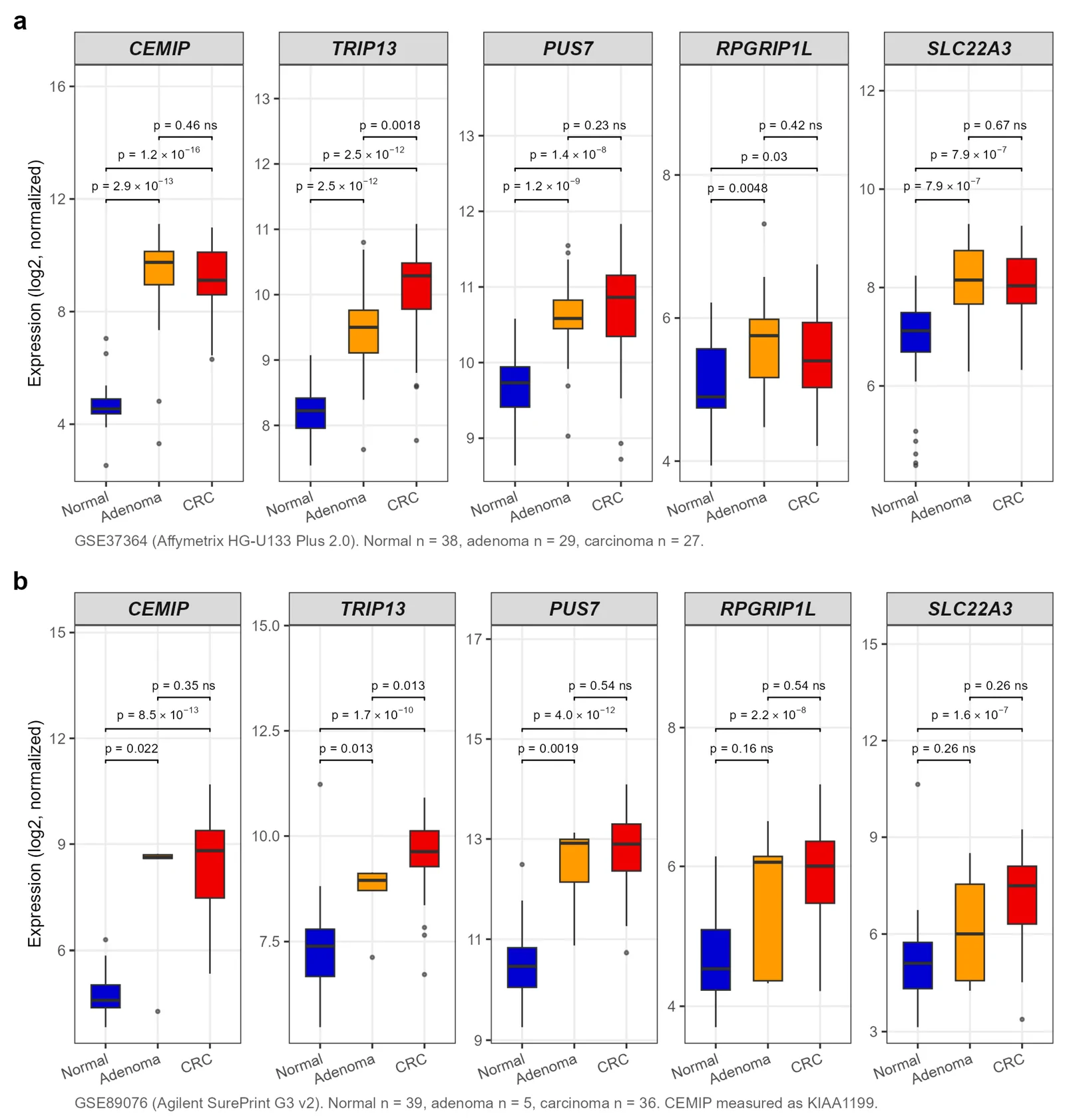
The convergent core in two adenoma series. Boxplots of log2 intensity for *CEMIP*, *TRIP13*, *PUS7*, *RPGRIP1L*, and *SLC22A3* in normal mucosa, adenoma, and carcinoma. (**a**) GSE37364 (Affymetrix HG-U133 Plus 2.0; 38 normal, 29 adenoma, 27 carcinoma; the two stage groups of the series pooled). (**b**) GSE89076 (Agilent SurePrint G3 v2; 39 normal, 5 adenoma, 36 carcinoma; *CEMIP* measured as *KIAA1199*). Brackets give BH-adjusted *p* from pairwise Wilcoxon tests; ns, adjusted *p* ≥ 0.05. These samples took no part in gene selection or in the model of Section 3.9, and adenomas were not entered into any classifier.

**Figure 13:**
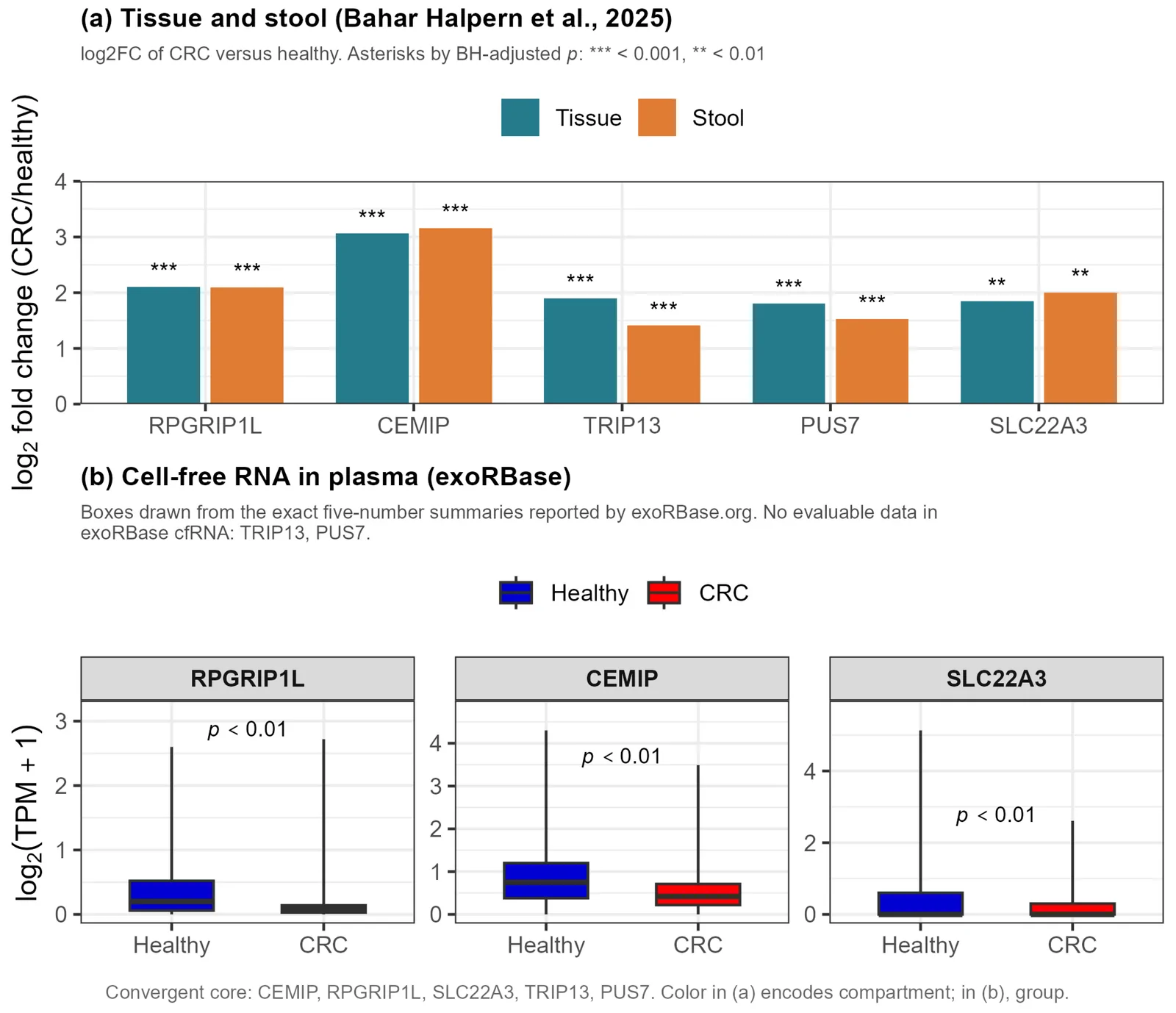
The convergent core in non-invasive specimens. (**a**) log_2_ fold change of colorectal cancer versus healthy controls in tumor tissue and in stool RNA-seq from Bahar Halpern et al. [25]; asterisks by BH-adjusted *p* as reported in that study (*** < 0.001, ** < 0.01). (**b**) Cell-free RNA in plasma from exoRBase; boxes are drawn from the five-number summaries reported by the database for healthy controls and patients, with the significance label reported there. *TRIP13* and *PUS7* had no evaluable data in exoRBase cell-free RNA. Both panels are descriptive; no classification metric was computed.

**Table 1:** Composition of the cohort by BioProject.

BioProject	Tumor (*n*)	Ref (*n*)	Total (*n*)	% of Total	Both Cond	LibrarySel	Layout	Platform
PRJNA1009137	48	0	48	46.2	No	cDNA	Paired	Illumina
PRJNA1055547	10	9	19	18.3	Yes	cDNA	Paired	Illumina
PRJNA596460	7	7	14	13.5	Yes	cDNA	Paired	Illumina
PRJNA913584	4	6	10	9.6	Yes	cDNA	Paired	Illumina
PRJNA394898	7	0	7	6.7	No	cDNA	Paired	Illumina
PRJNA603221	3	3	6	5.8	Yes	cDNA	Paired	Illumina
Total	79	25	104	100	4/6			

Tumor = colorectal tumor tissue; Ref = Non-tumor colorectal tissue; Cond = Condition; Sel = Selection. Library metadata are recorded in the SRA run table for each project.

**Table 2:** Route A candidate genes. Genes contributing to enriched terms, grouped by biological program and ordered by π-value within each program.

Gene	log_2_FC	Adjusted *p*	π-Value	Program	Representative	Representative Term	ES	Term-*p*
*TRIP13*	2.482973	1.49 × 10^−24^	59.16268	Proliferation	TRUE	Cell cycle	6.36791	5.10 × 10^−38^
*RFC3*	2.271097	1.36 × 10^−21^	47.38797	Proliferation	FALSE	DNA replication	7.2216	3.95 × 10^−24^
*PMAIP1*	2.145146	1.02 × 10^−22^	47.17900	Proliferation	FALSE	p53 signaling pathway	3.957201	3.35 × 10^−13^
*ATAD2*	1.857971	2.10 × 10^−23^	42.13615	Proliferation	FALSE	E2F TARGETS	13.19067	5.55 × 10^−10^
*CHEK1*	1.88962	3.77 × 10^−22^	40.48292	Proliferation	FALSE	Cell cycle	6.36791	5.10 × 10^−38^
*CAD*	1.482176	9.42 × 10^−27^	38.57511	Proliferation	FALSE	*MYC* TARGETS V1	9.994993	3.07 × 10^−8^
*CDK4*	1.614124	1.80 × 10^−22^	35.09813	Proliferation	FALSE	Cell cycle	6.36791	5.10 × 10^−38^
*CDK2*	1.401521	9.05 × 10^−25^	33.69694	Proliferation	FALSE	Mitotic G1 phase and G1/S transition	4.991065	6.10 × 10^−39^
*XPOT*	1.502704	4.57 × 10^−23^	33.57072	Proliferation	FALSE	Nucleocytoplasmic transport	3.375677	1.97 × 10^−14^
*MCM6*	1.479907	4.44 × 10^−23^	33.08012	Proliferation	FALSE	Cell cycle	6.36791	5.10 × 10^−38^
*CBX3*	1.298531	1.20 × 10^−25^	32.3613	Proliferation	FALSE	Polycomb repressive complex	3.077823	9.00 × 10^−10^
*NUP107*	1.306494	3.76 × 10^−25^	31.91147	Proliferation	FALSE	Nucleocytoplasmic transport	3.375677	1.97 × 10^−14^
*UTP20*	1.408868	1.45 × 10^−22^	30.76629	Proliferation	FALSE	*MYC* TARGETS V2	16.71576	1.53 × 10^−5^
*CBFB*	1.228292	2.60 × 10^−24^	28.96888	Proliferation	FALSE	*RUNX1* interacts with co-factors whose precise effect on *RUNX1* targets is not known	2.994639	7.89 × 10^−9^
*IPO7*	1.108502	5.42 × 10^−24^	25.79048	Proliferation	FALSE	Nucleocytoplasmic transport	3.375677	1.97 × 10^−14^
*CEMIP*	4.299795	5.09 × 10^−34^	143.1555	Nucleolus	TRUE	fibrillar center	2.861075	6.64 × 10^−6^
*NOP58*	1.491585	3.45 × 10^−24^	34.99517	Nucleolus	FALSE	rRNA processing in the nucleus and cytosol	2.789277	3.33 × 10^−22^
*PUS7*	1.953751	2.21 × 10^−28^	54.03342	tRNA	TRUE	tRNA processing	3.473979	5.42 × 10^−13^
*DKC1*	1.798246	4.23 × 10^−22^	38.43497	UPR	TRUE	Unfolded Protein Response	9.146361	2.53 × 10^−4^
*DDX10*	1.249454	4.67 × 10^−26^	31.65004	UPR	FALSE	Unfolded Protein Response	9.146361	2.53 × 10^−4^
*RPGRIP1L*	1.831627	1.79 × 10^−38^	69.13974	Ciliogenesis	TRUE	Cilium Assembly	2.247299	1.86 × 10^−12^
*CEP72*	2.283964	7.80 × 10^−22^	48.20953	Ciliogenesis	FALSE	Cilium Assembly	2.247299	1.86 × 10^−12^
*SLC22A3*	2.747256	1.92 × 10^−22^	59.66167	Solute transport	TRUE	SLC-mediated transmembrane transport	2.103828	9.89 × 10^−8^
*SLC6A6*	2.482293	7.14 × 10^−23^	54.97319	Solute transport	FALSE	SLC-mediated transmembrane transport	2.103828	9.89 × 10^−8^
*SLC39A10*	1.817281	1.34 × 10^−23^	41.56908	Solute transport	FALSE	SLC-mediated transmembrane transport	2.103828	9.89 × 10^−8^
*OSBPL3*	1.484941	7.94 × 10^−23^	32.81725	Nuclear membrane	TRUE	Nuclear membrane	2.469416	1.38 × 10^−7^

log_2_FC and adjusted *p*, DESeq2 with BioProject as covariate (apeglm); π-value = |log_2_FC| × −log_10_ (adjusted *p*); Program, biological program to which the gene was assigned (the one containing its most significant term); Representative, Yes for the gene with the highest π-value within its program; Representative term, most significant term of the gene within that program; ES, fold enrichment of the term; Term-*p*, adjusted *p* of the term (pathfindR, Bonferroni); adjusted *p*-values of the genes are Benjamini–Hochberg.

**Table 3:** The 26 candidate genes under the two batch-handling strategies.

Gene	Biological Program	log2FC A	Adjusted *p* A	log2FC B	Adjusted *p* B
*TRIP13*	Proliferation	2.48	1.5 × 10^−24^	2.45	4.4 × 10^−51^
*RFC3*	Proliferation	2.27	1.4 × 10^−21^	2.18	2.2 × 10^−40^
*PMAIP1*	Proliferation	2.15	1.0 × 10^−22^	2.07	4.8 × 10^−34^
*CHEK1*	Proliferation	1.89	3.8 × 10^−22^	1.77	1.4 × 10^−36^
*ATAD2*	Proliferation	1.86	2.1 × 10^−23^	1.83	3.0 × 10^−44^
*CDK4*	Proliferation	1.61	1.8 × 10^−22^	1.56	4.3 × 10^−31^
*XPOT*	Proliferation	1.50	4.6 × 10^−23^	1.43	1.1 × 10^−28^
*CAD*	Proliferation	1.48	9.4 × 10^−27^	1.45	6.0 × 10^−31^
*MCM6*	Proliferation	1.48	4.4 × 10^−23^	1.42	1.8 × 10^−30^
*UTP20*	Proliferation	1.41	1.5 × 10^−22^	1.42	2.5 × 10^−31^
*CDK2*	Proliferation	1.40	9.1 × 10^−25^	1.25	1.8 × 10^−25^
*NUP107*	Proliferation	1.31	3.8 × 10^−25^	1.28	2.1 × 10^−30^
*CBX3*	Proliferation	1.30	1.2 × 10^−25^	1.32	1.9 × 10^−31^
*CBFB*	Proliferation	1.23	2.6 × 10^−24^	1.09	1.1 × 10^−21^
*IPO7*	Proliferation	1.11	5.4 × 10^−24^	1.03	6.5 × 10^−20^
*CEMIP*	rRNA/nucleolus	4.30	5.1 × 10^−34^	4.25	3.8 × 10^−59^
*NOP58*	rRNA/nucleolus	1.49	3.5 × 10^−24^	1.43	1.9 × 10^−27^
*PUS7*	tRNA	1.95	2.2 × 10^−28^	1.95	4.5 × 10^−47^
*DKC1*	UPR	1.80	4.2 × 10^−22^	1.80	3.6 × 10^−37^
*DDX10*	UPR	1.25	4.7 × 10^−26^	1.25	1.5 × 10^−26^
*CEP72*	Ciliogenesis	2.28	7.8 × 10^−22^	2.00	3.3 × 10^−32^
*RPGRIP1L*	Ciliogenesis	1.83	1.8 × 10^−38^	1.97	1.2 × 10^−56^
*SLC22A3*	Solute transport	2.75	1.9 × 10^−22^	2.73	7.8 × 10^−43^
*SLC6A6*	Solute transport	2.48	7.1 × 10^−23^	2.43	5.4 × 10^−42^
*SLC39A10*	Solute transport	1.82	1.3 × 10^−23^	1.73	3.2 × 10^−40^
*OSBPL3*	Nuclear membrane	1.49	7.9 × 10^−23^	1.43	6.8 × 10^−29^

Values are the DESeq2 log2 fold change (apeglm) and the adjusted *p*-value for tumor versus reference tissue. Column A gives the result with BioProject as a covariate and column B the result on the ComBat-Seq-adjusted matrix. A gene is counted as agreeing when it keeps the same direction, an adjusted *p*-value below 0.05 and a log2 fold change of at least 1 in absolute value under both strategies.

**Table 4:** Genes retained by each route. Rows are the union of the seven Route A representatives and the twenty-one genes retained by Route B in all ten outer folds.

Gene	Route A Representative	Route B, 10/10 Folds	Route B Signature	Convergent Core
*RPGRIP1L*	✓	✓	✓	✓
*CEMIP*	✓	✓	—	✓
*PUS7*	✓	✓	—	✓
*SLC22A3*	✓	✓	—	✓
*TRIP13*	✓	✓	—	✓
*AJUBA*	—	✓	✓	—
*BMAL2 (ARNTL2)*	—	✓	✓	—
*ETV4*	—	✓	✓	—
*PPM1H*	—	✓	✓	—
*ATP11A*	—	✓	—	—
*CDH3*	—	✓	—	—
*DDIAS*	—	✓	—	—
*GTF2IRD1*	—	✓	—	—
*IFT56*	—	✓	—	—
*LRP8*	—	✓	—	—
*MTHFD1L*	—	✓	—	—
*NFE2L3*	—	✓	—	—
*SLC6A6*	—	✓	—	—
*SPTBN2*	—	✓	—	—
*TGFBI*	—	✓	—	—
*TNFSF15*	—	✓	—	—
*DKC1*	✓	—	—	—
*OSBPL3*	✓	—	—	—

✓, the gene belongs to that set; —, it does not. The convergent core is the intersection of the first two columns. *RPGRIP1L* is the only gene belonging to both final signatures. *ARNTL2* is the current HGNC symbol for *BMAL2*, the name used elsewhere in the text.

**Table 5:** Parameters of the convergent core classifier transferred to the external cohorts.

Gene	Internal Mean	Internal SD	β
*RPGRIP1L*	8.4303	0.9008	1.3074
*CEMIP*	10.4516	2.0001	1.088
*TRIP13*	8.9905	1.1769	1.0755
*SLC22A3*	8.9292	1.3818	1.0152
*PUS7*	9.5972	0.9832	0.7855
Intercept	—	—	2.4324

Coefficients apply to expression standardized with the internal mean and standard deviation given above; samples are classified as tumor at a predicted probability ≥ 0.50. In TCGA/GTEx, all parameters were applied unchanged. In GSE37364, only the coefficients were transferred: standardization used that cohort’s own mean and standard deviation, and the intercept was re-estimated within training folds.

## Data Availability

All data analyzed in this study are publicly available. The cohort was assembled from the NCBI Sequence Read Archive under accessions PRJNA1009137, PRJNA1055547, PRJNA596460, PRJNA913584, PRJNA394898, and PRJNA603221 and comprises 104 samples, 79 colorectal tumor and 25 non-tumor colorectal tissues; the contribution of each BioProject is given in Table 1. External tissue data were obtained from TCGA-COAD, TCGA-READ, and GTEx colon through recount3 and from Gene Expression Omnibus accession GSE37364. Stool comparisons used the published summary statistics of Bahar Halpern et al. (DOI 10.1038/s41598-025-17411-5), and plasma cell-free RNA values were taken from exoRBase 2.0 (DOI 10.1093/nar/gkab1085).

## Supplementary Materials

The following supporting information can be downloaded at: https://www.mdpi.com/article/10.3390/genes17101177/s1, Table S1: Overlap between published colorectal cancer signatures and the genes of the present study. Table S2: Leave-one-BioProject-out analysis of the 26 candidate genes. Figure S1: Protein–protein interaction network of the 26 candidate genes grouped into the seven biological programs of Table 2 (STRING v12.0, Homo sapiens, full network, minimum required interaction score 0.400, no additional interactors; line colors follow STRING evidence channels). Figure S2: Functional neighborhood of the convergent core (STRING v12.0, Homo sapiens, full network, minimum required interaction score 0.400, first shell limited to ten interactors, no second shell).

## Abbreviations

The following abbreviations are used in this manuscript:

AP	Average precision (area under the precision–recall curve)
AUC	Area under the receiver operating characteristic curve
BH	Benjamini–Hochberg
CI	Confidence interval
CRC	Colorectal cancer
FIT	Fecal immunochemical test
GEO	Gene Expression Omnibus
GTEx	Genotype-Tissue Expression project
KEGG	Kyoto Encyclopedia of Genes and Genomes
NPV	Negative predictive value
PPV	Positive predictive value
RNA-seq	RNA sequencing
ROC	Receiver operating characteristic
SRA	Sequence Read Archive
TCGA	The Cancer Genome Atlas
UPR	Unfolded protein response
VST	Variance-stabilizing transformation

## References

[1] Sung H., Filho A.M., Laversanne M., Ferlay J., Siegel R.L., Soerjomataram I., Jemal A., Bray F. (2026). Global Cancer Statistics 2024: GLOBOCAN Estimates of Incidence and Mortality Worldwide for 34 Cancers in 186 Countries. CA Cancer J. Clin..

[2] Espinosa-Tamez P., Suazo-Zepeda E., Sánchez-Blas H., Meneses-Medina M., Huitzil-Meléndez F.D., Van Loon K., Potter M.B., Lajous M. (2021). National and State-Level Colorectal Cancer Mortality Trends in Mexico, 1998–2018. Salud Publica Mex..

[3] Wen Q., Han S., Cui Y. (2025). Research Progress of Colorectal Cancer in Genomic and Transcriptomic at Multi-Level. Front. Genet..

[4] Kim Z., Lee J., Yoon Y.E., Yun J.W. (2024). Unveiling Prognostic RNA Biomarkers through a Multi-Cohort Study in Colorectal Cancer. Int. J. Mol. Sci..

[5] Chan S.C.H., Liang J.Q. (2022). Advances in Tests for Colorectal Cancer Screening and Diagnosis. Expert Rev. Mol. Diagn..

[6] González-Suárez B., Pagés M., Araujo I.K., Romero C., Rodríguez de Miguel C., Ayuso J.R., Pozo À., Vila-Casadesús M., Serradesanferm A., Ginès À. (2020). Colon Capsule Endoscopy versus CT Colonography in FIT-Positive Colorectal Cancer Screening Subjects: A Prospective Randomised Trial—The VICOCA Study. BMC Med..

[7] Imperiale T.F., Porter K., Zella J., Gagrat Z.D., Olson M.C., Statz S., Garces J., Lavin P.T., Aguilar H., Brinberg D. (2024). BLUE-CStudy Investigators Next-Generation Multitarget Stool DNATest for Colorectal Cancer Screening. N. Engl. J. Med..

[8] Seum T., Niedermaier T., Heisser T., Hoffmeister M., Brenner H. (2025). Next-Generation Multitarget Stool DNA vs. Fecal Immunochemical Test in Colorectal Cancer Screening. JAMA Intern. Med..

[9] Chung D.C., Gray D.M., Singh H., Issaka R.B., Raymond V.M., Eagle C., Hu S., Chudova D.I., Talasaz A., Greenson J.K. (2024). A Cell-Free DNA Blood-Based Test for Colorectal Cancer Screening. N. Engl. J. Med..

[10] Leinonen R., Sugawara H., Shumway M. (2011). International Nucleotide Sequence Database Collaboration. The Sequence Read Archive. Nucleic Acids Res..

[11] Zhang Y., Parmigiani G., Johnson W.E. (2020). ComBat-Seq: Batch Effect Adjustment for RNA-Seq Count Data. NAR Genom. Bioinform..

[12] Love M.I., Huber W., Anders S. (2014). Moderated Estimation of Fold Change and Dispersion for RNA-Seq Data with DESeq2. Genome Biol..

[13] Nygaard V., Rødland E.A., Hovig E. (2016). Methods That Remove Batch Effects While Retaining Group Differences May Lead to Exaggerated Confidence in Downstream Analyses. Biostatistics.

[14] Ahluwalia P., Kolhe R., Gahlay G.K. (2021). The Clinical Relevance of Gene Expression Based Prognostic Signatures in Colorectal Cancer. Biochim. Biophys. Acta Rev. Cancer.

[15] Salazar R., Roepman P., Capella G., Moreno V., Simon I., Dreezen C., Lopez-Doriga A., Santos C., Marijnen C., Westerga J. (2011). Gene Expression Signature to Improve Prognosis Prediction of Stage II and III Colorectal Cancer. J. Clin. Oncol..

[16] Agesen T.H., Sveen A., Merok M.A., Lind G.E., Nesbakken A., Skotheim R.I., Lothe R.A. (2012). ColoGuideEx: A Robust Gene Classifier Specific for Stage II Colorectal Cancer Prognosis. Gut.

[17] Guinney J., Dienstmann R., Wang X., de Reyniès A., Schlicker A., Soneson C., Marisa L., Roepman P., Nyamundanda G., Angelino P. (2015). The Consensus Molecular Subtypes of Colorectal Cancer. Nat. Med..

[18] Shi M., Beauchamp R.D., Zhang B. (2012). A Network-Based Gene Expression Signature Informs Prognosis and Treatment for Colorectal Cancer Patients. PLoS ONE.

[19] Maurya N.S., Kushwaha S., Chawade A., Mani A. (2021). Transcriptome Profiling by Combined Machine Learning and Statistical R Analysis Identifies TMEM236 as a Potential Novel Diagnostic Biomarker for Colorectal Cancer. Sci. Rep..

[20] Maurya N.S., Kushwaha S., Vetukuri R.R., Mani A. (2023). Unlocking the Potential of the CA2, CA7, and ITM2C Gene Signatures for the Early Detection of Colorectal Cancer: A Comprehensive Analysis of RNA-Seq Data by Utilizing Machine Learning Algorithms. Genes.

[21] Michiels S., Koscielny S., Hill C. (2005). Prediction of Cancer Outcome with Microarrays: A Multiple Random Validation Strategy. Lancet.

[22] Ein-Dor L., Zuk O., Domany E. (2006). Thousands of Samples Are Needed to Generate a Robust Gene List for Predicting Outcome in Cancer. Proc. Natl. Acad. Sci. USA.

[23] Venet D., Dumont J.E., Detours V. (2011). Most Random Gene Expression Signatures Are Significantly Associated with Breast Cancer Outcome. PLoS Comput. Biol..

[24] Wilks C., Zheng S.C., Chen F.Y., Charles R., Solomon B., Ling J.P., Imada E.L., Zhang D., Joseph L., Leek J.T. (2021). recount3: Summaries and Queries for Large-Scale RNA-Seq Expression and Splicing. Genome Biol..

[25] Bahar Halpern K., Kent I., Yakubovsky O., Ben-Moshe S., Barkai T., Fine M., Novoselsky R., Israeli A., Nachmany I., Itzkovitz S. (2025). Stool Shed Cell Transcriptomics Mirrors Tumor Biology and Enables Colorectal Cancer Diagnosis. Sci. Rep..

[26] Liao Y., Smyth G.K., Shi W. (2019). The R Package Rsubread Is Easier, Faster, Cheaper and Better for Alignment and Quantification of RNA Sequencing Reads. Nucleic Acids Res..

[27] Liao Y., Smyth G.K., Shi W. (2014). featureCounts: An Efficient General Purpose Program for Assigning Sequence Reads to Genomic Features. Bioinformatics.

[28] Zhu A., Ibrahim J.G., Love M.I. (2019). Heavy-Tailed Prior Distributions for Sequence Count Data: Removing the Noise and Preserving Large Differences. Bioinformatics.

[29] Liberzon A., Birger C., Thorvaldsdóttir H., Ghandi M., Mesirov J.P., Tamayo P. (2015). The Molecular Signatures Database Hallmark Gene Set Collection. Cell Syst..

[30] Szklarczyk D., Kirsch R., Koutrouli M., Nastou K., Mehryary F., Hachilif R., Gable A.L., Fang T., Doncheva N.T., Pyysalo S. (2023). The STRING Database in 2023: Protein-Protein Association Networks and Functional Enrichment Analyses for Any Sequenced Genome of Interest. Nucleic Acids Res..

[31] Xiao Y., Hsiao T.H., Suresh U., Chen H.I., Wu X., Wolf S.E., Chen Y. (2014). A Novel Significance Score for Gene Selection and Ranking. Bioinformatics.

[32] Friedman J., Hastie T., Tibshirani R. (2010). Regularization Paths for Generalized Linear Models via Coordinate Descent. J. Stat. Softw..

[33] Pedregosa F., Varoquaux G., Gramfort A., Michel V., Thirion B., Grisel O., Blondel M., Prettenhofer P., Weiss R., Dubourg V. (2011). Scikit-Learn: Machine Learning in Python. J. Mach. Learn. Res..

[34] Ritchie M.E., Phipson B., Wu D., Hu Y., Law C.W., Shi W., Smyth G.K. (2015). limma Powers Differential Expression Analyses for RNA-Sequencing and Microarray Studies. Nucleic Acids Res..

[35] Davis S., Meltzer P.S. (2007). GEOquery: A Bridge between the Gene Expression Omnibus (GEO) and BioConductor. Bioinformatics.

[36] Lai H., Li Y., Zhang H., Hu J., Liao J., Su Y., Li Q., Chen B., Li C., Wang Z. (2022). exoRBase 2.0: An Atlas of mRNA, lncRNA and circRNA in Extracellular Vesicles from Human Biofluids. Nucleic Acids Res..

[37] Sheng N., Yan L., Wu K., You W., Gong J., Hu L., Tan G., Chen H., Wang Z. (2018). TRIP13 Promotes Tumor Growth and Is Associated with Poor Prognosis in Colorectal Cancer. Cell Death Dis..

[38] Hua Q., Zhang B., Xu G., Wang L., Wang H., Lin Z., Yu D., Ren J., Zhang D., Zhao L. (2021). CEMIP, a Novel Adaptor Protein of OGT, Promotes Colorectal Cancer Metastasis through Glutamine Metabolic Reprogramming via Reciprocal Regulation of β-Catenin. Oncogene.

[39] Ko Y.G., Jo J.H., Song S.Y., Lee H.S. (2025). The Crucial Role of CEMIP in Cancer Metastasis: Mechanistic Insights and Clinical Implications. FASEB J..

[40] Dong B., Wang B., Fan M., Zhang J., Zhao Z. (2024). Comprehensive Analysis to Identify PUS7 as a Prognostic Biomarker from Pan-Cancer Analysis to Osteosarcoma Validation. Aging.

[41] Hohmann T., Hohmann U., Dehghani F., Grisk O., Jasinski-Bergner S. (2025). Identification and Characterization of the RNA Modifying Factors PUS7 and WTAP as Key Components for the Control of Tumor Biological Processes in Renal Cell Carcinomas. Curr. Issues Mol. Biol..

[42] Gu J., Dong D., Long E., Tang S., Feng S., Li T., Wang L., Jiang X. (2019). Upregulated OCT3 Has the Potential to Improve the Survival of Colorectal Cancer Patients Treated with (m)FOLFOX6 Adjuvant Chemotherapy. Int. J. Colorectal Dis..

[43] Lin Y.W., Yan M.D., Shih Y.L., Hsieh C.B. (2009). The Basal Body Gene, RPGRIP1L, Is a Candidate Tumor Suppressor Gene in Human Hepatocellular Carcinoma. Eur. J. Cancer.

[44] Guo J., Yang Y., Ji Z., Yao M., Xia X., Sha X., Huang M. (2021). Case Report: Novel RPGRIP1L Gene Mutations Identified by Whole Exome Sequencing in a Patient with Multiple Primary Tumors. Front. Genet..

[45] O’Connell M.J., Lavery I., Yothers G., Paik S., Clark-Langone K.M., Lopatin M., Watson D., Baehner F.L., Shak S., Baker J. (2010). Relationship between Tumor Gene Expression and Recurrence in Four Independent Studies of Patients with Stage II/III Colon Cancer Treated with Surgery Alone or Surgery plus Adjuvant Fluorouracil plus Leucovorin. J. Clin. Oncol..

[46] Lee C.Y., Pan H.J., Lee Y.C., Nguyen H.D., Ni Y.C., Yee K.X., Nguyen M.T., Wu Y.F., Lee K.H. (2026). Age-Stratified Transcriptomic Profiling Identifies CEMIP as a Candidate Biomarker in Early-Onset Colorectal Cancer and Reveals an Association with PTCH1-Related Hedgehog Signaling. Genes.

[47] Hou P.F., Shi P., Jiang T., Yin H., Chu S., Shi M., Bai J., Song J. (2020). DKC1 Enhances Angiogenesis by Promoting HIF-1α Transcription and Facilitates Metastasis in Colorectal Cancer. Br. J. Cancer.

[48] Wang C., He Y., He Y., Liang W., Zhou C., Wu M., Meng Z., Li W., Cao J. (2023). Prognostic and Biological Function Value of OSBPL3 in Colorectal Cancer Analyzed by Multi-Omic Data Analysis. BMC Gastroenterol..

[49] Jiang T., Liu X., Liu H., Du K., Wang Y., Fan H., Zhang Y., Cui L., Zhang H., Zhang C. (2025). ETV4 Interacts with LOXL2 to Induce Epigenetic Activation of NID1 during Colorectal Cancer Progression. Int. J. Biol. Sci..

[50] Fu D., Zhang M., Cai M., Wang M., Huang Z., Lin Z., Liu Y., Qian W., Chen G., Liang Y. (2026). ETV4 Promotes Colorectal Cancer Progression by Reprogramming Asparagine Metabolism to Remodel the Stromal Microenvironment. Adv. Sci..

[51] Fonseca A.S., Ramão A., Bürger M.C., de Souza J.E.S., Zanette D.L., de Molfetta G.A., de Araújo L.F., de Barros e Lima Bueno R., Aguiar G.M., Placa J.R. (2021). ETV4 Plays a Role on the Primary Events during the Adenoma-Adenocarcinoma Progression in Colorectal Cancer. BMC Cancer.

[52] Zhang L., Zhang F., Liang H., Qin X., Mo Y., Chen S., Xie J., Hou X., Deng J., Hao E. (2025). Hippo/YAP Signaling Pathway in Colorectal Cancer: Regulatory Mechanisms and Potential Drug Exploration. Front. Oncol..

[53] Mohammadpour S., Torshizi Esfahani A., Sarpash S., Vakili F., Zafarjafarzadeh N., Mashaollahi A., Pardakhtchi A., Nazemalhosseini-Mojarad E. (2024). Hippo Signaling Pathway in Colorectal Cancer: Modulation by Various Signals and Therapeutic Potential. Anal. Cell. Pathol..

[54] Mazzoccoli G., Pazienza V., Panza A., Valvano M.R., Benegiamo G., Vinciguerra M., Andriulli A., Piepoli A. (2012). ARNTL2 and SERPINE1: Potential Biomarkers for Tumor Aggressiveness in Colorectal Cancer. J. Cancer Res. Clin. Oncol..

[55] Yang J., Lin D., Huang Y., Yin S., Chen M., Sun H., Zhu W., Chen E., Deng Y., Zhao E. (2025). Clock Gene ARNTL2 Enhances 5-Fluorouracil Resistance in Colon Cancer by Upregulating SLC7A11 to Suppress Ferroptosis. Redox Biol..

[56] Sugiura T., Noguchi Y., Sakurai K., Hattori C. (2008). Protein Phosphatase 1H, Overexpressed in Colon Adenocarcinoma, Is Associated with CSE1L. Cancer Biol. Ther..

[57] Xu X., Zhu L., Yang Y., Pan Y., Feng Z., Li Y., Chang W., Sui J., Cao F. (2019). Low Tumor PPM1H Indicates Poor Prognosis in Colorectal Cancer via Activation of Cancer-Associated Fibroblasts. Br. J. Cancer.

[58] Miyoshi N., Ishii H., Mimori K., Tanaka F., Nagai K., Uemura M., Sekimoto M., Doki Y., Mori M. (2010). ATP11A Is a Novel Predictive Marker for Metachronous Metastasis of Colorectal Cancer. Oncol. Rep..

[59] Yazlovitskaya E.M., Graham T.R. (2023). Type IV P-Type ATPases: Recent Updates in Cancer Development, Progression, and Treatment. Cancers.

[60] Wang Z., Xue M., Liu J., Jiang H., Li F., Xu M., Wang H. (2024). ATP11A Promotes Epithelial-Mesenchymal Transition in Gastric Cancer Cells via the Hippo Pathway. J. Cancer.

[61] Yasunaga M., Matsumura Y. (2014). Role of SLC6A6 in Promoting the Survival and Multidrug Resistance of Colorectal Cancer. Sci. Rep..

[62] Cao T., Zhang W., Wang Q., Wang C., Ma W., Zhang C., Ge M., Tian M., Yu J., Jiao A. (2024). Cancer SLC6A6-Mediated Taurine Uptake Transactivates Immune Checkpoint Genes and Induces Exhaustion in CD8+ T Cells. Cell.

