# Supplementary material for: Two Transcriptomic Analyses Converge on a Five-Gene Core for Colorectal Cancer Discrimination: External Validation in Independent Tissue Cohorts
Source: Genes (Basel). 2026 Sep 24;17(10):1177. doi: 10.3390/genes17101177 (PMC13704336; doi:10.3390/genes17101177)
Supplement: Supplementary file 1 [file genes-17-01177-s001.zip › FigureS1_String.pdf]

Figure S1

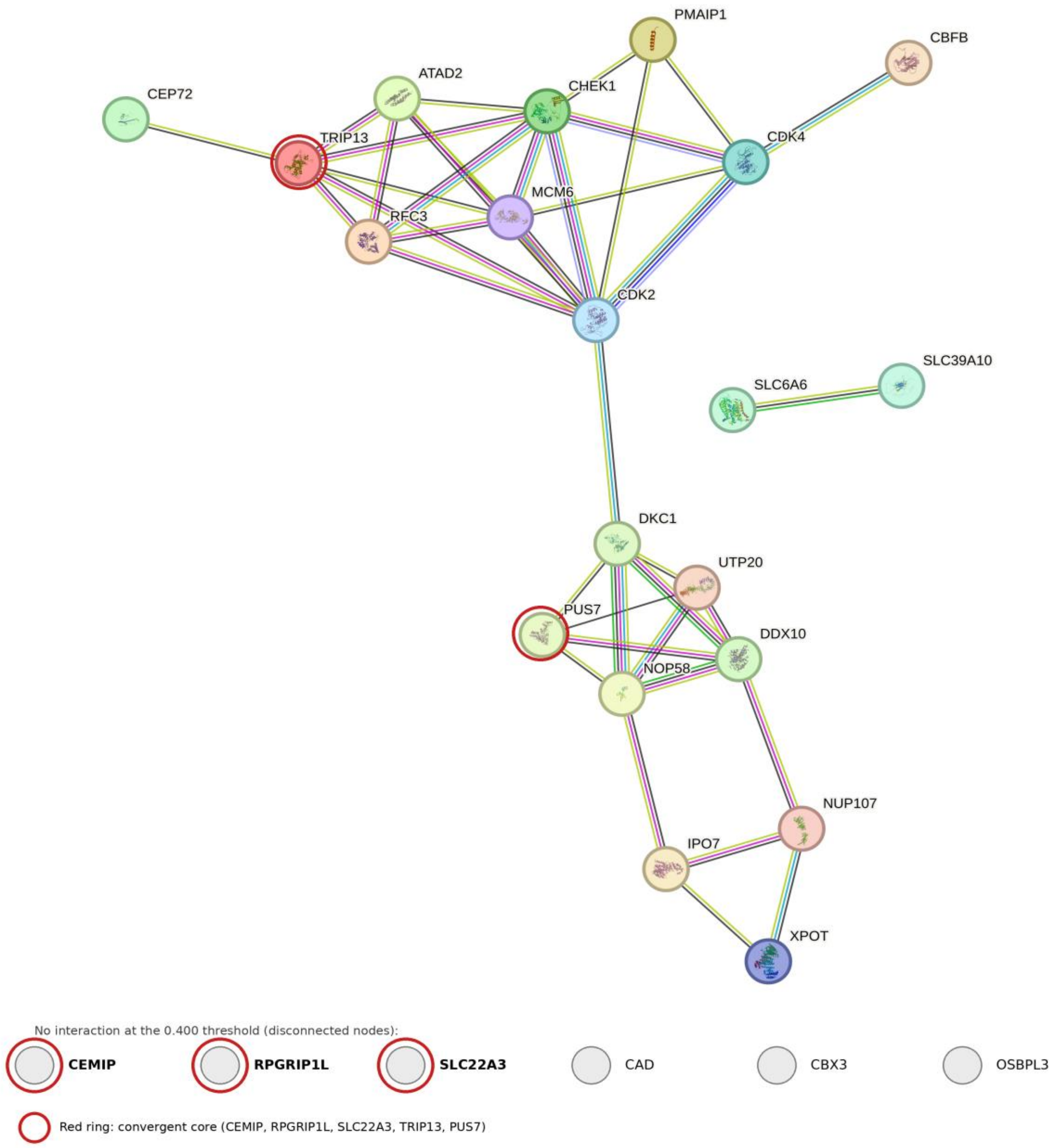

Figure S1. Protein–protein interaction network of the 26 candidate genes grouped into the seven biological programs of Table 2 (STRING v12.0, Homo sapiens, full network, minimum required interaction score 0.400, no additional interactors; line colors follow STRING evidence channels). Forty interactions were retrieved among 20 of the 26 proteins, 15 of them with combined score ≥ 0.700. Two components follow the programs: one of proliferation and cell-cycle genes and one of rRNA-processing, tRNA-modification and nuclear-transport genes, joined by a single CDK2–DKC1 edge (0.601); SLC6A6 and SLC39A10 form a separate pair. Red rings mark the convergent core: TRIP13 (six interactions, 0.42–0.68) and PUS7 (four, 0.41–0.83) sit inside those components, while CEMIP, RPGRIP1L and SLC22A3 have no interaction with any other candidate at this threshold and are shown below the network with the other disconnected nodes (CAD, CBX3, OSBPL3). The five core genes were reached by discrimination rather than by network membership.
