# Supplementary material for: Two Transcriptomic Analyses Converge on a Five-Gene Core for Colorectal Cancer Discrimination: External Validation in Independent Tissue Cohorts
Source: Genes (Basel). 2026 Sep 24;17(10):1177. doi: 10.3390/genes17101177 (PMC13704336; doi:10.3390/genes17101177)
Supplement: Supplementary file 1 [file genes-17-01177-s001.zip › FigureS2_STRING_core.pdf]

Figure S2

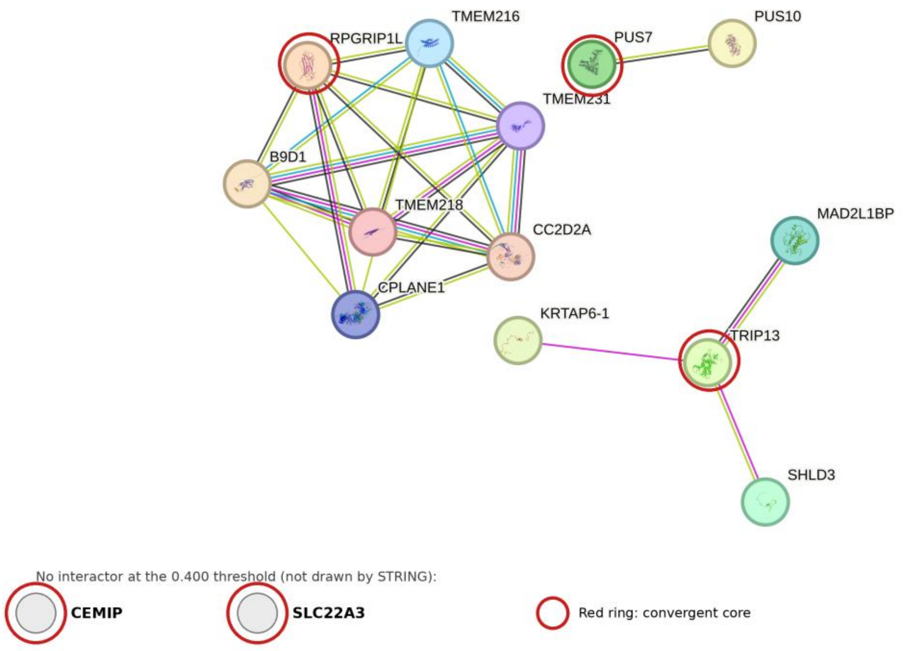

Figure S2. Functional neighborhood of the convergent core (STRING v12.0, Homo sapiens, full network, minimum required interaction score 0.400, first shell limited to ten interactors, no second shell). The five core genes (red rings) are shown with the ten proteins most connected to them: 15 nodes and 24 edges, with line colors following STRING evidence channels. Six interactors belong to RPGRIP1L and form the MKS complex of the ciliary transition zone (B9D1, CC2D2A, CPLANE1, TMEM216, TMEM218, TMEM231; scores 0.57–0.97), three to TRIP13 (MAD2L1BP 0.997, SHLD3 0.917, KRTAP6-1 0.487) and one to PUS7 (PUS10 0.825). CEMIP and SLC22A3 have no interactor at this threshold and are not drawn by STRING; they are shown below the network. No interactor links two core genes. Functional enrichment of the 15-node network is accordingly dominated by ciliary terms (ciliary transition zone, GO:0035869, 6 of 71 proteins, FDR 2.9×10^{-8}; MKS complex, GO:0036038, 4 of 13, FDR 5.3×10^{-7}) with tRNA pseudouridine synthesis (GO:0031119, 2 of 6, FDR 0.018) contributed by PUS7 and PUS10; these terms describe the neighbors of two core genes rather than the core as a set.
