# Supplementary material for: Two Transcriptomic Analyses Converge on a Five-Gene Core for Colorectal Cancer Discrimination: External Validation in Independent Tissue Cohorts
Source: Genes (Basel). 2026 Sep 24;17(10):1177. doi: 10.3390/genes17101177 (PMC13704336; doi:10.3390/genes17101177)
Supplement: Supplementary file 1 [file genes-17-01177-s001.zip › TableS1_published_signatures_overlap.pdf]

Table S1. Overlap between published colorectal cancer signatures and the genes of the present study. Overlap was assessed by gene symbol against the 26 Route A candidates (Table 2), the convergent core (CEMIP, RPGRIP1L, SLC22A3, TRIP13, PUS7) and the Route B signature (RPGRIP1L, ETV4, BMAL2, PPM1H, AJUBA). No signature was re-trained on the present data. The three recurrence signatures were built to predict outcome after resection rather than to separate tumor from mucosa, and their gene lists were taken from the secondary sources indicated because the primary articles are not open access; symbols are given as current HGNC names, with the original name in parentheses where it differs.

Signature	Purpose	Genes (n)	Gene list	Source of the list	Shared with the 26 Route A candidates or the convergent core	Shared with the Route B signature
**ColoPrint (Salazar et al., 2011) [REF]**	Recurrence risk, stage II-III colon cancer	18 (17 gene symbols and one unannotated probe)	MCTP1, LAMA3, CTSC, PYROXD1, EDEM1, IL2RB, ZNF697, SLC6A11, IL2RA, CYFIP2, PIM3, LIF, PLIN3, HSD3B1, ZBED4, PPARA, THNSL2, CA438802	List as reported in Lin et al., 2017 [REF, VERIFICAR]	None	None
**ColoGuideEx (Agesen et al., 2012) [REF]**	Recurrence risk, stage II colorectal cancer	13	AZGP1, BNIP3, DSC3, ENPP3, EPHA7, KLK6, SEMA3A, SESN1, CXCL10, CXCL13, MMP3, PIGR, TUBA1B	List as reported in Park et al., 2013 [REF, VERIFICAR]	None	None
**Oncotype DX Colon (O'Connell et al., 2010) [REF]**	Recurrence score, stage II-III colon cancer	12 (7 recurrence genes and 5 reference genes)	BGN, MYC, FAP, GADD45B, INHBA, MKI67, MYBL2; reference ATP5F1E (ATP5E), GPX1, PGK1, UBB, VDAC2	List as reported in Clark-Langone et al., 2010 [REF, VERIFICAR]	None	None
**Maurya et al., 2021 [REF]**	Tumor versus normal, diagnostic	5 (2 final)	VSTM2A, NR5A2, TMEM236, GLDN, ETFDH (final: VSTM2A, TMEM236)	Primary article	None	None
**Maurya et al., 2023 [REF]**	Tumor versus normal, early detection	3	CA2, CA7, ITM2C	Primary article	None	None
**Lee et al., 2026 [REF]**	Candidate genes in early-onset colorectal cancer	10 evaluated (1 prioritized)	DPEP1, KRT80, FABP6, NKD2, FOXQ1, CEMIP, ETV4, TESC, FUT1, GAS2 (prioritized: CEMIP)	Primary article	CEMIP (Route A candidate and convergent core)	ETV4 (Route B signature)
