# Supplementary material for: Two Transcriptomic Analyses Converge on a Five-Gene Core for Colorectal Cancer Discrimination: External Validation in Independent Tissue Cohorts
Source: Genes (Basel). 2026 Sep 24;17(10):1177. doi: 10.3390/genes17101177 (PMC13704336; doi:10.3390/genes17101177)
Supplement: Supplementary file 1 [file genes-17-01177-s001.zip › TableS2_leave_one_bioproject_out.pdf]

Table S2. Leave-one-BioProject-out analysis of the 26 candidate genes. For each exclusion, DESeq2 was refitted on the remaining samples with the same model (~ BioProject + Condition), the same low-count filter (≥ 10 counts in ≥ 3 samples) and apeglm shrinkage. Values are log2 fold change for tumor versus reference tissue. All 156 gene-by-exclusion values kept a positive direction and adjusted $p < 0.05$; an asterisk marks the single value below the magnitude threshold (|log2FC| ≥ 1). † Convergent core.

Gene	without PRJNA1009137	without PRJNA1055547	without PRJNA596460	without PRJNA913584	without PRJNA394898	without PRJNA603221
Tumor / reference remaining	31 / 25	69 / 16	72 / 18	75 / 19	72 / 25	76 / 22
TRIP13 †	2.50	2.22	2.57	2.61	2.49	2.43
RFC3	2.30	1.72	2.41	2.49	2.28	2.35
PMAIP1	2.14	2.33	1.89	2.23	2.15	2.12
ATAD2	1.87	1.70	1.72	2.02	1.86	1.89
CHEK1	1.90	1.57	1.99	1.99	1.90	1.91
CAD	1.48	1.19	1.51	1.62	1.48	1.52
CDK4	1.61	1.37	1.58	1.76	1.61	1.65
CDK2	1.40	1.51	1.21	1.46	1.41	1.44
XPOT	1.50	1.41	1.51	1.57	1.50	1.47
MCM6	1.48	1.27	1.50	1.58	1.48	1.49
CBX3	1.30	1.10	1.40	1.34	1.30	1.30
NUP107	1.31	1.19	1.22	1.41	1.31	1.35
UTP20	1.42	1.21	1.40	1.52	1.42	1.44
CBFB	1.23	1.26	1.07	1.30	1.23	1.24
IPO7	1.11	1.09	0.96 *	1.24	1.11	1.11
CEMIP †	4.32	4.04	4.04	4.59	4.30	4.33
NOP58	1.49	1.29	1.41	1.63	1.49	1.54
PUS7 †	1.96	1.69	1.95	2.10	1.95	1.97
DKC1	1.81	1.54	1.75	1.97	1.80	1.83
DDX10	1.24	1.01	1.29	1.36	1.26	1.27
RPGRIP1L †	1.82	1.49	1.89	1.98	1.83	1.88
CEP72	2.28	1.92	2.08	2.54	2.29	2.40
SLC22A3 †	2.75	2.68	2.51	2.98	2.75	2.71
SLC6A6	2.48	2.53	2.28	2.53	2.48	2.53
SLC39A10	1.81	1.63	1.73	1.93	1.82	1.87
OSBPL3	1.49	1.36	1.52	1.51	1.48	1.50

Per exclusion: without PRJNA1009137: 36349 genes after filtering, 7441 DEGs (3249 overexpressed), 26/26 candidates and 5/5 core genes meeting all three criteria; without PRJNA1055547: 35741 genes after filtering, 4536 DEGs (2329 overexpressed), 26/26 candidates and 5/5 core genes meeting all three criteria; without PRJNA596460: 36845 genes after filtering, 5903 DEGs (2542 overexpressed), 25/26 candidates and 5/5 core genes meeting all three criteria; without PRJNA913584: 35843 genes after filtering, 7160 DEGs (3223 overexpressed), 26/26 candidates and 5/5 core genes meeting all three criteria; without PRJNA394898: 37356 genes after filtering, 6736 DEGs (2996 overexpressed), 26/26 candidates and 5/5 core genes meeting all three criteria; without PRJNA603221: 36433 genes after filtering, 6733 DEGs (3067 overexpressed), 26/26 candidates and 5/5 core genes meeting all three criteria.
